# Effectiveness of Caregiver‐Mediated Spoken Language Interventions for Children Under Five at Risk of Developmental Language Disorder: A Systematic Review and Meta‐Analysis

**DOI:** 10.1111/1460-6984.70304

**Published:** 2026-08-21

**Authors:** Victoria Johnstone‐Cooke, Marion Rutherford, Ainuska Sheripkanova, Rachel Hutcheson, Ann Hodson

**Affiliations:** ^1^ Clinical Audiology Speech and Language Research Centre Queen Margaret University Edinburgh Scotland; ^2^ Institute for Global Health and Development Queen Margaret University Edinburgh Scotland; ^3^ NHS Lothian Edinburgh Scotland

**Keywords:** caregiver‐mediated language intervention, developmental language disorder, early language development, early language intervention, parent‐mediated language intervention

## Abstract

**Background and Aims:**

Caregiver‐mediated interventions are commonly used by Speech and Language Therapists to support early language development. Developmental Language Disorder (DLD) is associated with reduced quality of life throughout the lifespan. Understanding factors that predict intervention success is essential for developing appropriate, cost‐effective therapy provision for the approximately 12% of preschool children who present with early markers for Developmental Language Disorder (DLD). This systematic review and meta‐analysis examined the effectiveness of caregiver‐mediated spoken language interventions for under‐fives at risk of DLD, and factors influencing intervention effectiveness.

**Methods:**

A systematic review following PRISMA guidelines was conducted. Five electronic databases were searched to identify experimental studies comparing caregiver‐mediated spoken language interventions to control conditions in under‐fives presenting with risk factors for DLD. Risk factors included prematurity, socioeconomic factors, caregiver language development concerns, and formal or informal language screening or assessment scores. Twenty‐six experimental studies with 1407 child participants were included in qualitative synthesis. Meta‐analysis was performed on nine Randomised Controlled Trials involving 947 children.

**Results:**

Effectiveness was examined for outcomes including child language gains, child wellbeing, inclusion and attainment. Meta‐analysis indicated a significant effect of caregiver‐mediated spoken language interventions on language outcomes compared to treatment‐as‐usual, non‐language intervention or waitlist control conditions. Non‐language outcomes were evaluated via qualitative synthesis. Interventions significantly improved language development trajectories for under‐fives presenting with risk factors or early markers for DLD.

**Conclusion and Implications:**

This review contributes to the growing evidence base demonstrating that caregiver‐mediated interventions can positively impact language development and wellbeing outcomes for children under five at risk of DLD. These findings support the implementation of caregiver‐mediated environmental language interventions in clinical practice to maximise accessibility and cost‐effectiveness while delivering optimal outcomes for vulnerable populations.

**WHAT THIS PAPER ADDS:**

*What is already known on this subject*

- Previous research on caregiver‐mediated spoken language interventions has highlighted gaps in the evidence regarding the impact of risk factors, demographic characteristics, dosage and intervention components on child language outcomes. Developmental Language Disorder has relatively high population prevalence, estimated at 7%. Prevalence is associated with risk factors including low household socioeconomic status (SES), prematurity and late language emergence. In contrast to its prevalence, there is low public and professional awareness of DLD and a low diagnostic rate. Therefore, a strengthened evidence base and additional insights into the factors affecting success of family‐based interventions is important in order to increase the effectiveness of service provision and care planning for this underserved population. Timely and effective intervention with young children presenting with early markers for DLD has the potential to offer lifelong improvement to their wellbeing, inclusion and attainment outcomes.
- Recent systematic reviews of the effectiveness of caregiver‐mediated language interventions had differences in population age range and diagnostic inclusion criteria.

*What this paper adds to existing knowledge*

- Our review examines the effectiveness of caregiver‐mediated early spoken language interventions on child language, attainment and wellbeing, and on caregiver self‐efficacy and adherence to language support strategies. Our population was children under five presenting with risk factors for Developmental Language Disorder, in the absence of other neurodevelopmental or genetic conditions such as intellectual disability or autism. This review adds depth and detail to the evidence base supporting the effectiveness of caregiver‐mediated spoken language interventions in improving outcomes for this population of young children, and factors that influence their success.

*What are the potential or actual clinical implications of this work?*

- The high prevalence of Developmental Language Disorder, estimated at around 7% of the population, and the strong association with risk factors including low SES, prematurity and late language emergence, coupled with the low awareness of DLD and low diagnostic rate, mean that a strengthened evidence base and additional insights into the factors affecting success of family‐based interventions can increase the effectiveness of service provision and care planning for this population. Timely and effective intervention in this group of young children has the potential to improve wellbeing and attainment outcomes across the lifespan. This review contributes to our understanding of how to implement cost‐effective, socially valid and maximally engaging partnership working with families of young children at risk for DLD.

## 1 Introduction

### 1.1 Definition and Terminology

Developmental Language Disorder, or DLD, is a lifelong difficulty with understanding and speaking one's native language, which creates barriers to communication, learning and attainment (Bishop et al. 2016). DLD is defined as a persistent deficit in one or more areas of language acquisition, which impacts on the individual's communication and learning, and which does not resolve by school age (Bishop et al. 2017; Conti‐Ramsden et al. 2018). DLD markers are present from early childhood, and diagnostic exclusion criteria specify that the disorder cannot be explained by sensory, motor, neurological, intellectual, or biomedical conditions, nor by other neurodevelopmental conditions such as autism (American Psychiatric Association 2013; Bishop et al. 2016; World Health Organization 2022). DLD is associated with lower quality of life in childhood (Eadie et al. 2018) and throughout the lifespan (Ziegenfusz et al. 2022). DLD can affect both receptive and expressive ability, and can present across a range of areas, including phonology, grammar, word‐finding, narrative and pragmatic skills (Bishop et al. 2016; RCSLT 2020). Spoken language is a fundamental means for children to express their needs, interact with others socially, play and learn (Snow et al. 2021).

In a UK population‐based survey, Norbury et al. (2016) found that 7.58% of children had language abilities significantly below age expectation without any known cause, and a further 2.34% presented with language difficulties associated with another condition. This is consistent with previous prevalence studies using the older terminology of Specific Language Impairment (Beitchman et al. 1986; Tomblin et al. 1997). Thus, in real‐world terms, two or more children in every school classroom of 30 experience persistent and pervasive difficulty with language at a level that would meet diagnostic criteria for DLD (Norbury et al. 2016). An even larger minority of pre‐schoolers present with markers for DLD but do not yet meet diagnostic criteria; between the ages of two and five, up to 12% of children present with spoken language skills significantly below age expectations, where this is not attributable to any identifiable primary cause such as hearing impairment, cognitive delay or social communication difference (Reilly et al. 2023).

Low rates of DLD diagnosis make it difficult to investigate the long‐term efficacy of different supports (Calder et al. 2022). This disparity between public awareness and actual prevalence becomes evident when contrasted with autism, which is more widely recognised despite its lower occurrence (2% compared with 7.5%) (Bishop et al. 2012; Kim et al. 2023; Thordardottir et al. 2021).

### 1.2 Risk Factors for DLD

A number of neurobiological, environmental and genetic factors are associated with an increased risk of Developmental Language Disorder. Premature birth is also associated with elevated risk of DLD (Valavani et al. 2022), and a wider body of evidence links preterm birth with lower language skills in the preschool years (e.g. Sentenac et al. 2020; Zerbeto et al. 2015).

Children from families experiencing socioeconomic disadvantage are more likely to present with early markers for DLD, and these are more likely to persist until school age (Law et al. 2017). When socio‐economic status (SES) is stratified at population level, a significant difference has been found between different groups’ language outcomes. At five years old, clinically significant language difficulties are seen in between three and 10% of children in the highest quintile of socioeconomic status compared to 18% to 23% in the lowest (Sante et al. 2022), with some studies placing the prevalence of primary language impairment in the most deprived areas at 65% or higher (Norbury et al. 2021). There is also a negative effect on awareness of and access to services for families experiencing socioeconomic disadvantage (Dockrell et al. 2022; Gibbard and Smith 2016; Morgan et al. 2017).

The relationship between socioeconomic status and DLD remains a complex and contested area of developmental linguistics. Some authors argue that low SES has been disproportionately emphasised in etiological frameworks of DLD, potentially obscuring other significant factors (Norbury et al. 2021). Beginning with Hart et al. (1997), a series of studies (Goldin‐Meadow et al. 2014; Schwab et al. 2016) have linked disparities in language exposure, quality of interaction and language learning opportunities to socioeconomic deprivation. More recently, authors have drawn attention to the wide variability of child language skills within socio‐economic strata, suggesting that the relationship between socioeconomic status and DLD maybe more nuanced than previously thought (Law et al. 2022; Masek et al. 2021). Further, Allen and Spencer (2022) criticise the simplistic pathologisation of language environments in lower SES homes, and Rakhlin et al. (2024) offer evidence that genetic and socio‐economic risk factors can be successfully dissociated using sensitive assessment tools. Both SES and language ability are complex and multifactorial constructs, and many factors that confer risk of DLD interact with SES, for example, parent education level and adverse childhood experiences (Dockrell et al. 2022), meaning that a straightforward causality model is difficult to establish (Pace et al. 2017).

A large‐scale Australian study (Calder et al. 2022) found no evidence of sex differences in DLD prevalence rates, in contrast to previous estimates that males were 1.3 to 1.5 times more likely to be diagnosed than females (Bishop et al. 2017). However, referral bias in males may lead to a lower number of girls with DLD being identified (Morgan et al. 2017).

### 1.3 Caregiver‐Mediated Early Language Intervention

Children learn language through positive interactions with familiar adults who act as language models in naturally occurring environments (Hoyne et al. 2022; Lanjekar et al. 2022). Training or coaching caregivers to deliver optimal early language support to their children offers a cost‐effective means of increasing the dosage of environmental interventions compared to practitioner‐delivered therapy alone (O'Toole et al. 2021). Strategies for promoting language development are incorporated into day‐to‐day communication and play. Interventions include a focus on increasing caregiver responsiveness, providing more frequent successful communication opportunities via adapting everyday play and activity of daily living, modelling and expanding on children's utterances and modifying the quantity and quality of caregivers’ language input (Roberts et al. 2011).

Caregiver‐mediated early language interventions have typically employed either a naturalistic language teaching environment (Heidlage et al. 2020) or dialogic reading (Sawyer et al. 2025) to stimulate spoken language. In naturalistic language teaching environment interventions, strategies are based on the theory that linguistic development is rooted in a development of joint attention that is, a situation where both communication partners have attention on the same object while this is named verbally by one communication partner (Adamson et al. 2020). Responsive interaction strategies include following the child's lead in play and communication, and recasting, which involves allowing the child to hear their own utterances modelled back by an adult communication partner with additional verbal and grammatical information added. Other strategies include modelling, asking and answering questions during interactive book‐reading, pausing to offer an opportunity for the child to take a conversational turn, and emphasising key words while reinforcing their meaning with objects, natural gesture and facial expression (Heidlage et al. 2020). Dialogic book reading employs similar responsiveness strategies with additional language‐supportive asking and answering of questions during interactive book sharing (Hammer et al. 2016).

### 1.4 Effectiveness of Interventions

In this review, we use the term effectiveness to refer to the performance of interventions under typical, real‑world conditions, where contextual constraints, resource limitations, and routine practice variability shape outcomes (Nilsen et al. 2025). Effectiveness differs from efficacy, which concerns performance under controlled or ideal circumstances. Pooled effect sizes in quantitative synthesis are considered to reflect the effectiveness of interventions.

In terms of measurable outcomes for inclusion in the review, we considered that a broad range of linguistic and non‑linguistic child outcomes, as well as caregiver outcomes reflecting increased confidence and understanding, would provide the most comprehensive range of holistic indicators of effectiveness for the evidence synthesis. Accordingly, the review included primary language‐based outcomes such as scores on caregiver‑reported observation checklists, researcher‑coded observation schedules, caregiver‑ and researcher‑reported checklists capturing the frequency and range of specified communication behaviours, and scores on formal language assessment tools, alongside secondary outcomes including caregiver‑reported confidence in supporting language development, and child educational attainment, well‑being and inclusion, early literacy skills, phonological skills, and phonological working memory.

We also considered that factors associated with effectiveness, and with the lasting effect of interventions, were pertinent to the research question.

### 1.5 Importance of Early Intervention

There has been recent discussion of the value of viewing language development as a public health issue (McKean et al. 2023; Neiling and Cutshaw 2023). Having DLD substantially increases the risk of adverse outcomes in educational attainment, employment success, health and psychosocial well‐being (Dubois et al. 2020; Le et al. 2021; World Health Organization 2021). Early childhood is a critical period of neuroplasticity and rapid cognitive development. Language learning occurs most rapidly and effectively during this period (McKean et al. 2017). This means that early interventions to support language development are particularly important in ameliorating the effects of DLD and its lifelong impact on attainment, participation and wellbeing (Chang 2017; Conti‐Ramsden et al. 2018; McLeod et al. 2019). Early identification of individuals at risk, followed by targeted support provision, can act as a valuable protective factor in the lives of young children (Goh et al. 2021). Identifying effective supports for Developmental Language Disorder therefore has potential to have a significant positive impact on affected individuals and the wider society. The RCSLT's research prioritisation exercise (2019) placed evidence‐based interventions for DLD among the top three research priorities for the Speech and Language Therapy profession in the UK, emphasising the need for cost‐effective approaches that maximise impact and promote equity of access.

### 1.6 Previous Studies

Three earlier systematic reviews have offered meta‐analyses of the effectiveness of caregiver‐mediated spoken language intervention. The meta‐analysis by Heidlage et al. (2020) found that parent‐implemented language interventions significantly improve children's expressive vocabulary, with play and routine‐based approaches showing larger average effects (*g* = 0.50) than shared book reading interventions (*g* = 0.37), though play‐based interventions demonstrated much greater variability in outcomes. The substantial variation in play‐based intervention results suggested that their effectiveness may depend on specific characteristics of the child population or how the intervention is delivered. Roberts et al. (2011) found that parent training programmes demonstrated moderate positive effects on children's communication, engagement, and language outcomes (*g* = 0.33), with particularly strong effects on parents' use of language support strategies (*g* = 0.55). Children with Developmental Language Disorder showed the greatest benefits, with large effect sizes for both receptive (*g* = 0.92) and expressive language (*g* = 0.83), while children at risk for language impairments exhibited moderate improvements in receptive language and engagement. Roberts et al. (2019) undertook a systematic review and meta‐analysis with a broader focus, exploring the association of caregiver training with language development in children, including typically developing populations. They also found that caregiver‐led language interventions positively influenced language development in children with or at risk of language difficulties, showing moderate effects on child outcomes and strong effects on parent behaviours.

### 1.7 Rationale for this Review

The current review utilises study data from the intervening time period to add to current understanding about the effectiveness of caregiver‐mediated spoken language interventions. During this time, further RCTs (McGowan et al. 2024; Nielsen et al. 2023) and quasi‐experimental studies (e.g., Suttora et al. 2021; Tang et al. 2024; Zuccarini et al. 2020) with relatively large participant populations have added to the evidence base. Our review also differs in several key characteristics from previous work. Firstly, the age range is limited in this study to children under 5. Secondly, this review is focused on caregiver‐mediated spoken language interventions only. Thirdly, participant criteria were restricted to children with primary language difficulties, that is, no known attributable cause of their communication difficulty, for example autism or hearing loss.

Therefore, the purpose of this systematic review is to update and expand current understanding of the effectiveness of caregiver‐mediated spoken language intervention on language attainment and well‐being outcomes for children under five who present with indications and/or risk factors that are associated with later meeting diagnostic criteria for Developmental Language Disorder.

## 2 Methods

This review was registered in the PROSPERO database of systematic review protocols and follows Preferred Reporting Items for Systematic Reviews and Meta Analysis guidelines (Page et al. 2021). The registration number was CRD42024540158.

Research questions:

What is the effectiveness of caregiver‐mediated spoken language interventions on child language, attainment and wellbeing outcomes in children under five at risk of developmental language disorder?
What factors are associated with greater effectiveness of caregiver‐mediated spoken language interventions?

### 2.1 Search Strategy

A systematic database search was conducted for experimental and quasi‐experimental study designs. The primary reviewer conducted the searches using a predetermined search strategy, published at: https://www.crd.york.ac.uk/PROSPEROFILES/540158_STRATEGY_20240429.pdf. Victoria Johnstone‐Cooke, Rachel Hutcheson and Ainuska Sheripkanova took part in preliminary test screening and scoping. Victoria Johnstone‐Cooke developed the finalised search strategy and wrote the registered protocol for publication. Victoria Johnstone‐Cooke and Ann Hodson screened 299 identified papers at Title and Abstract stage, excluding 173 irrelevant studies, and a further 36 papers were screened at Full‐text Review stage by Victoria Johnstone‐Cooke, Rachel Hutcheson and Ann Hodson. Any disagreements were resolved by discussion between the authors. Searches were conducted in the following electronic databases: PubMed, EBSCOhost incorporating CINAHL Plus, ERIC, MEDLINE and PsycINFO databases, Scopus, Web of Science and Speechbite. Key papers’ reference lists were individually searched to identify further potentially relevant studies. Publication dates were limited to 2013 and later, and papers written in any language were included. Searches were conducted on 1st May 2024, and re‐run on 13th January 2025 to identify any newly published studies for inclusion. Data extraction was conducted by the Victoria Johnstone‐Cooke. 10% of the data was randomly checked for accuracy by Ann Hodson and Marion Rutherford. Data extracted included sample size, participant demographics, DLD risk factor, intervention format and duration, outcome measures, and effect sizes. Authors were contacted by email in the case of missing data.

### 2.2 Inclusion and Exclusion Criteria

Studies conducted with both randomised and non‐randomised research designs were included in the qualitative synthesis of results. The studies with RCT designs were additionally included in the meta‐analysis. One RCT (Peredo et al. 2022) did not report the mean and standard deviation of results data, which was required to calculate the standardised mean difference for this synthesis, and authors were not able to provide this data within the timescale of this study. This RCT was excluded from meta‐analysis. Results of other study designs were included in qualitative synthesis.

Inclusion criteria for this review are defined in Table 1 below:

**TABLE 1:** Inclusion criteria for this review.

	Inclusion criteria	Exclusion criteria
Population	Children under 5 years of age Presenting with risk factors for Developmental Language Disorder including delayed expressive language, family socioeconomic stressors, prematurity, behavioural difficulties, or family history of speech, language and communication needs Any region	Studies specifically addressing bilingualism Children with chromosomal, metabolic, progressive or acquired cognitive/neurological disorders Children with deafness or hearing loss Children with an existing diagnosis of intellectual disability Children with a diagnosis of autism or where the stated primary difficulty is social communication
Intervention	Caregiver‐ or primary caregiver‐mediated oral language intervention delivered indirectly via healthcare professionals (e.g., Speech and Language Therapists, Speech and Language Therapy Assistants) involving face‐to‐face or online, individual or group caregiver coaching in techniques to promote language development in everyday life	Intervention delivered directly by healthcare or education professionals Behavioural or wellbeing interventions Universal/targeted written advice or signposting only, without an individualised coaching and modelling component
Comparator/ Control	No intervention, intervention unrelated to language development, ‘wait and see’ approaches, universal/targeted advice only, or ‘treatment as usual’ without a stated caregiver‐mediated component	Healthcare or education professional delivered direct intervention ‘Treatment as usual’ with a defined caregiver‐mediated component
Outcome	Language assessment gains Child participation, wellbeing or quality of life Educational attainment and cognitive development Caregiver self‐efficacy or adherence to strategies	Economic effects, efficiency, or cost of service delivery Caregiver quality of life or caregiver stress Caregiver engagement or retention in intervention

For the purposes of this review, we defined caregivers as the main provider of home‐based care. Many studies used the term ‘parent‐mediated’, and these studies were included in the review, however we felt that ‘caregiver’ offered a more inclusive terminology reflecting diverse family structures, and caregivers in both family and institutional home environments.

We included children under the age of five because five years of age is the currently accepted cut‐off for the youngest reliable diagnosis of DLD (Bishop et al. 2016), and we were interested in the effectiveness of the interventions in question on children presenting with risk factors for DLD, rather than those already presenting with a clear diagnosis.

For the purposes of this review, naturalistic language teaching environment interventions, dialogic reading interventions, and interventions that combined elements of both, were judged to meet inclusion criteria.

### 2.3 Study Quality Appraisal

Due to the anticipated heterogeneity of study designs and methodologies, the Modified Downs and Black Methodological Quality Checklist (Downs and Black 1998) was selected for study quality appraisal. This is one of two quality appraisal tools recommended by the Cochrane Collaboration for appraising the quality of diverse study designs including non‐randomised studies (Chandler et al. 2019). Using this checklist, studies were assigned a grade of ‘excellent’ (24–28 points), ‘good’ (19–23 points), ‘fair’ (15–18 points) or ‘poor’ (14 points or below). Non‐controlled studies could score a maximum of 21 points using this tool. Checklist questions, incorporating the modification first reported in Reed et al. (2014), are reported in Appendix 1.

The methodological quality of all included studies was independently assessed by Victoria Johnstone‐Cooke and Rachel Hutcheson. Following independent appraisal, ratings were compared and any discrepancies were resolved through discussion until consensus was reached. Arbitration by Ann Hodson was available but was not required.

#### 2.3.1 Ethical Approval

Institutional ethical approval is not required for conducting systematic reviews. However, PRISMA guidelines (Page et al. 2021) were adhered to in our review.

Study characteristics are summarised in Table 2.

**TABLE 2:** Characteristics of included studies.

Study Country	Study design	Population (N) (Intervention/Control)	DLD risk factor	Intervention (Control)	Mode of delivery Delivered by	Outcomes	Outcome measures	Quality/27
(Akemoglu et al. 2022) USA	Single‐case multiple baseline design	**3** (n/a)	Language delay and/or developmental delay in Individualized Family Service Plan (IFSP)	**Parent‐Implemented Communication Strategies– Storybook (i‐PiCSS)** (n/a)	**Online** Research assistant	Language assessment gains (observational/ informal assessment); Caregiver adherence to strategies	Informal observational measures of child communication behaviours; Observational coding of caregiver fidelity to and rate of strategy use	Fair (15)
(Bagner et al. 2016) USA	RCT	**60** (31/29)	Behavioural difficulties	**Infant Behaviour Programme** (Standard paediatric care)	**Face to face** Research assistant	Informal language assessment gains	Child Language Data Exchange System (CHILDES) (MacWhinney 2000): Total utterances (tokens), Different utterances (types)	Good (20)
(Burgoyne et al. 2018) United Kingdom	RCT	**208** (103/105)	Socioeconomic factors (targeted areas)	**Parent‐mediated early language enrichment programme** (Movement skills and self‐care programme)	**Face to face** Trained children's centre staff	Formal and informal language assessment gains	CELF Preschool II UK—Expressive Vocabulary and Sentence Structure subtests (Semel et al. 2006); Renfrew Action Picture Test (APT)(Renfrew 2003); BPVS3 – British Picture Vocabulary Scale, 3rd Ed.(L. Dunn et al. 2009); York Assessment of Reading for Comprehension (YARC) stories (Snowling et al. 2009) (adapted) informal observational measure of story retelling; York Assessment of Reading Comprehension (YARC)—Early Word Recognition (Snowling et al. 2012)	Excellent (24)
(Buschmann et al. 2015) Germany	RCT	**43** (23/20)	Language concern reported by caregiver / Screening tool (MacArthur Bates)	**Heidelberg Parent‐Based Language Intervention (HPLI)** (Treatment as usual with direct SLT)	**Face to face** (not recorded)	Formal language assessment	Expressive Vocabulary Test for 3–5‐Year‐Olds (AWST‐R) (Kiese‐Himmel 2005); SETK 3‐5 Plural Forming, Sentence Comprehension, Word Span, Non‐word Repetition, Sentence Repetition subtests (Grimm 2001); K‐ABC Number Recall, Word Order subtests (Kaufman et al. 1983)	Excellent (26)
(Cunningham et al. 2019) Canada	Case series	**24** (n/a)	Language concern reported by caregiver	**Target Word, The Hanen Program for Parents of Children who are Late Talkers**	**Face to face** Speech and Language Therapist	Formal and Informal language assessment gains, Consonant inventory	FOCUS‐34 (Focus on the Outcomes of Communication Under Six) (Thomas‐Stonell et al. 2012); Communication Function Classification System (CFCS) (Hidecker et al. 2017); MacArthur–Bates Communicative Development Inventories–Words & Gestures (MBCDI) (Fenson et al. 2006) Informal checklist assessing consonant inventory	Fair (16)
(Cycyk et al. 2020) USA	Case series	**8** (n/a)	Language concern reported by caregiver	**Language and Play Every Day (LAPE)**	**Face to face** Student research assistants supervised by SLTs	Informal language assessment gains Parent self‐efficacy/confidence in supporting language development	MacArthur–Bates Communicative Development Inventories (MCDI): Words and Gestures (Spanish and English versions) (Fenson et al. 2006; Jackson‐Maldonado et al. 2021); Systematic coding of caregiver use of communication‑ facilitating strategies, child rate of communication, child rate of initiation; Caregiver survey	Fair (16)
(Falkus et al. 2016) United Kingdom	Case series	**18** (n/a)	Language concern reported by caregiver	**Parent–child interaction therapy (PCIT)**	**Face to face** Speech and Language Therapist	Formal and Informal language assessment gains	Child Mean Length of Utterance; Observational scale of caregiver interaction strategies (e.g., commenting, waiting, praising); Ratio of child to parent speech	Fair (18)
(McDonald et al. 2019) United Kingdom	Case series	**7** (n/a)	Language concern reported by caregiver	**Home Talk intervention**	**Face to face** Speech and Language Therapy assistant	Formal language assessment gains	Language Use Inventory (LUI)—Words subscale (O'Neill 2009)	Poor (13)
(McGowan et al. 2024) USA	RCT	**64** (32/33)	Prematurity	**Caregiver‐mediated early language enrichment programme** (Infant safety programme)	**Face to face** Research assistant	Formal language assessment gains, Cognitive skill gains	Bayley Scales of Infant and Toddler Development, Third Edition (BSID‐III) (Bayley 2006)	Excellent (25)
(Mettler et al. 2023) USA	Feasibility study	**5** (n/a)	Language concern reported by caregiver	**Caregiver‐implemented telehealth model of Vocabulary Acquisition and Usage for Late Talkers (VAULT)**	**Online** Speech and Language Therapist	Formal language assessment, Caregiver confidence, engagement with programme, adherence to strategies	MacArthur–Bates Communicative Development Inventories (CDI): Words & Sentences (Spanish and English versions) (Fenson et al. 2006; Jackson‐Maldonado et al. 2021); Caregiver interviews and surveys	Fair (17)
(Moore et al. 2014) USA	Case series	**8** (n/a)	Standardised language score	**Language and Play Everyday (LAPE)**	**Face to face** Speech and Language Therapist	Formal and informal language assessment	Preschool Language Scale—Fourth Edition (PLS‐4) (Zimmerman et al. 2002); MacArthur–Bates Communicative Development Inventory (CDI‑II) (Fenson et al. 2006); Child Mean Length of Utterance; Frequency of child communication behaviours from coded video samples	Fair (16)
(Nielsen et al. 2023) United Kingdom	RCT	**102** (52/50)	Socioeconomic factors (e.g., targeted areas)	**Talking Together** (Waitlist control, usual care and input reported)	**Face to face** Language Development Workers	Formal language assessment gains	Oxford Communication Development Inventory—Short (CDI‐Short) (Hamilton et al. 2000); WellComm Language Assessment (Sandwell Primary Care Trust 2012)	Fair (18)
(Olson et al. 2016) USA	Case series	**31** (n/a)	Socioeconomic factors (e.g., targeted areas)	**uTALK program: Text messaging programme promoting parent mediated language development strategies** (n/a)	**Text message** Fully automated via text	Parent self‐efficacy/confidence in supporting language development	Parent surveys and interviews	Fair (18)
(Peredo et al. 2022) USA	Intent‐to‐Treat RCT	**20** (10/10)	Language concern reported, Standardised language score	**EMT en Español via Teach‐Model‐Coach‐Review approach** (Waitlist control)	**Face to face** One SLT, one researching Psychotherapist	Formal language assessment; Caregiver adherence to strategies	Manualised coding of caregiver uses of strategies during interactions	Good (23)
(Quinn et al. 2021) USA	Case series	**4** (n/a)	Standardised language score	**Enhanced Milieu Teaching (EMT)** (n/a)	**40% in‐person and 60% telepractice sessions** Speech and Language Therapist	Informal language assessment gains; Caregiver adherence to strategies	Count measures of child communication acts, Number of different words; Coding of caregiver implementation fidelity and frequency of strategy use	Fair (16)
(Rajesh and Venkatesh 2019) India	Case series	**9** (n/a)	Language concern reported, Standardised language score	**Low intensity caregiver‐mediated language program** (n/a)	**Face to face** Not reported	Informal language assessment gains; Caregiver adherence to strategies; Caregiver confidence in supporting language development	Language Assessment Tool (LAT) derived from Subba Rao (1992); Caregiver‐reported child language behaviour change; Coding of caregiver‐child interaction videos; Caregiver questionnaires	Fair (15)
(Roberts et al. 2014) USA	Case series	**4** (n/a)	Standardised language score	**Teach‐Model‐Coach‐Review instructional approach** (n/a)	**Face to face** Research assistant	Formal and informal language assessment gains; Caregiver adherence to strategies	Preschool Language Scale—Fourth Edition (PLS‐4) (Zimmerman et al. 2002); Expressive One‐Word Picture Vocabulary Test—Fourth Edition (EOWPVT‐4) (Brownell 2000); Manualised coding of child Mean Length of Utterance, number of different words and total number of words; Coding of caregiver behaviour from video‐recorded sessions	Fair (15)
(Roberts and Kaiser 2015) USA	RCT	**97** (45/52)	Standardised language score	**Enhanced Milieu Teaching (EMT)** (Waitlist control, usual care and input reported)	**Face to face** Research assistant	Formal and Informal language assessment gains; Caregiver adherence to strategies	Preschool Language Scale—Fourth Edition (PLS‐4) (Zimmerman et al. 2002); Peabody Picture Vocabulary Test—Fourth Edition (PPVT‐4) (L. M. Dunn et al. 2007); MacArthur–Bates Communicative Development Inventories (CDI): Words & Sentences (Fenson et al. 2006); Expressive One‐Word Picture Vocabulary Test—Fourth Edition (EOWPVT‐4) (Brownell 2000); Observational coding of frequency of caregiver use of strategies	Excellent (24)
(Sawyer et al. 2025) USA	RCT	**31** (16/15)	**Individualised Educational Plan and working diagnosis of DLD**	**Parents Plus** (Waitlist control, usual care and input reported)	**Online** Research assistant	Formal and informal language assessment gains	CELF Preschool–Second Edition (CELF Preschool–2) Core Language Index (Semel et al. 2006); Manualised coding of child Mean Length of Utterance, number of different words and total number of words; Researcher‐created probe for morphosyntactic skills	Good (19)
(Senent‐Capuz et al. 2021) Spain	Quasi‐experimental study	**17** (n/a)	Language concern reported, Standardised language score	**It Takes Two to Talk—The Hanen Program for Parents (ITTT)** (Clinician‐directed therapy)	**Face to face** Speech and Language Therapist	Formal and informal language assessment gains	MacArthur–Bates Communicative Development Inventories (MCDI‐S) (Spanish version) (Mariscal 2007); Reynell Developmental Language Scales (RDLS‐III) (Edwards et al. 1999); Parent Perception of Language Development (PPOLD) (Romski et al. 2000)	Fair (16)
(Suttora et al. 2021) Italy	Quasi‐experimental study	**46** (n/a)	Prematurity	**Caregiver‐implemented dialogic reading and focused stimulation techniques** (Waitlist control)	**Face to face** Psychologist	Formal language assessment gains	MacArthur–Bates Communicative Development Inventories (CDI): Words & Sentences (Fenson et al. 2006); Manualised coding of child Mean Length of Utterance, word types and word tokens	Good (20)
(Tang et al. 2024) China	Case series	**146** n/a	Language concern reported, Standardised language score	**Online parent training program: Bethel Hearing and Speaking Training Center Family Training for Early Communication & Language Development (Bethel Family Training Program, BFT)** (n/a)	**Online** Speech and Language Therapist	Formal language assessment	Gesell Developmental Schedules (GDS) (Zhang et al. 1994)	Fair (17)
(Vally et al. 2015) South Africa	RCT	**91** (49/42)	Socioeconomic factors (e.g., targeted areas)	**Dialogic book‐sharing programme** (Waitlist control)	**Face to face** Trained community members	Formal and informal language assessment gains	MacArthur–Bates Communicative Development Inventories (CDI) (translated into isiXhosa by study team) (Fenson et al. 2006); Adapted receptive language measure modelled on Peabody Picture Vocabulary Test—Revised (PPVT‑R) (L. M. Dunn et al. 2007)	Excellent (24)
(Vrinda and Kunnath 2023) India	Case series	**8** (n/a)	Language Disorder provisional diagnosis received	**Caregiver‐mediated early language enrichment programme** (n/a)	**Face to face** Speech and Language Therapist	Formal language assessment	Assessment of Language Development (ALD)—receptive and expressive language (Lakkanna et al. 2008); Get Ready to Read (GRTR) (Benmeleh 2011)	Poor (13)
(Wake et al. 2015) Australia	RCT	**301** (158/143)	Language concern reported	**Let's Learn Language: modified ‘You Make the Difference’ programme** (Treatment as usual)	**Face to face** Three interventionists (one with a speech pathology background and two with psychology backgrounds)	Formal and informal language assessment gains	Preschool Language Scale—Fourth Edition (PLS‐4)—receptive and expressive (Zimmerman et al. 2002); MacArthur–Bates Communicative Development Inventories (MCDI‐III) (Fenson et al. 2006) Sure Start language measure (Roy et al. 2005)	Excellent (25)
(Zuccarini et al. 2020) Italy	Non‐randomised controlled trial	**50** (27/23)	Language concern reported	**Caregiver‐implemented dialogic book reading intervention (Girolametto)** (Waitlist control)	**Face to face** Psychologist	Informal language assessment gains, Cognitive scores	MacArthur–Bates Communicative Development Inventories (CDI) (Italian version) (Caselli et al. 2015); Bayley Scales of Infant and Toddler Development, Third Edition (BSID‐III) (Bayley 2006)	Fair (17)

OpenMeta[Analyst] software (Wallace et al. 2012) was utilised for data synthesis and statistical analyses. Statistical significance was set at *p* < 0.05. Assessment of continuous variables utilised the mean difference. Results were presented in forest plots with 95% confidence intervals (CIs).

### 2.4 Heterogeneity of Studies

Both Cochran's Q test and the I^2^ statistic were used to assess heterogeneity across studies in the meta‐analysis. Cochran's Q test is a statistical method for evaluating heterogeneity in meta‐analyses by testing whether differences between studies exceed what would be expected by chance alone. It operates under the null hypothesis that all studies share the same true effect size, with a significant *p*‐value indicating meaningful heterogeneity (Greenland et al. 2012). I^2^ is a statistical measure that expresses the percentage of total variation across studies in a meta‐analysis that is attributable to actual differences between trials rather than to sampling error (Higgins et al. 2002). Cochran's Q test expresses the statistical significance of heterogeneity across studies, while I^2^ quantifies its magnitude, allowing contextualization of results. Interpretation of the I^2^ results categorised low heterogeneity as 0%–25%, moderate heterogeneity as 25%–75%, and considerable heterogeneity as 75%–100%. Inverse variance was used in calculations where the I^2^ was moderate or high.

## 3 Results

**FIGURE 1:**
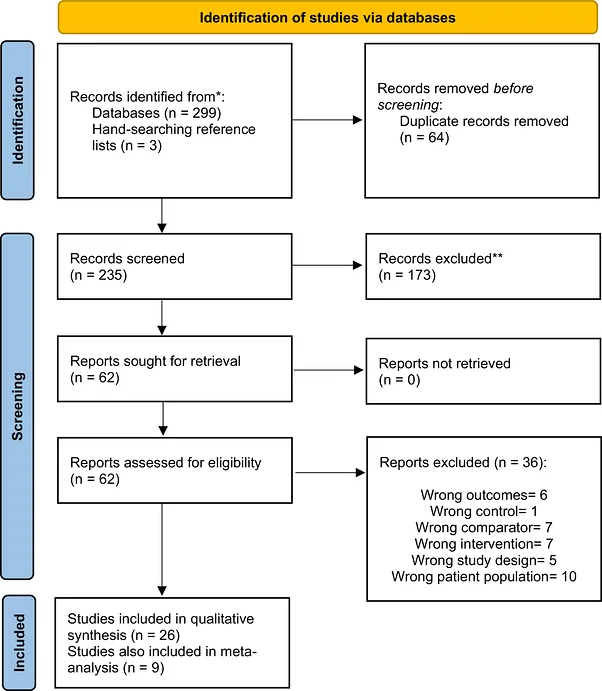
PRISMA Flow chart of study selection (from Moher et al. 2009).

Twenty‐six intervention studies with a total of 1405 participants met inclusion criteria for the review and were included in the qualitative synthesis. Of these, nine RCTs with a total participant population of 997 were also included in meta‐analysis. Participants ranged from 23 weeks to 42 months in age. Of the studies, ten were randomised controlled trials (RCTs) with a total population of 1017, one was a non‐randomised controlled trial, and the remaining 15 were quasi‐experimental studies including case series and single‐case designs. Three studies (Suttora et al. 2021; Vally et al. 2015; Zuccarini et al. 2020) used dialogic reading interventions, with the remaining 23 studies employing naturalistic language learning strategies in everyday home activities. A PRISMA flow chart of study selection is illustrated in Figure 1.

### 3.1 Study Quality Appraisal

The 26 included studies demonstrated variable methodological quality according to the modified Downs and Black checklist, with scores ranging from 13 to 26 (mean = 18.73, SD = 3.98) out of 28 possible points. Overall quality ratings comprised six studies rated Excellent (23%), four Good (15%), fourteen Fair (54%), and two Poor (8%). Study quality appraisal is shown in Table 3.

**TABLE 3:** Quality appraisal of studies.

Question	1	2	3	4	5	6	7	8	9	10	11	12	13	14	15	16	17	18	19	20	21	22	23	24	25	26	27	Total	Rating
**Study and year**																													
Akemoglu et al. 2022	Y	Y	Y	Y	**P**	Y	N	Y	Y	N	/	/	Y	N	N	Y	Y	Y	Y	Y	/	/	N	N	N	Y	N	15	Fair
Bagner et al. 2016	Y	Y	Y	Y	**Y**	Y	Y	Y	Y	N	/	/	Y	N	N	Y	Y	Y	Y	Y	/	Y	Y	N	Y	Y	Y	21	Good
Burgoyne et al. 2018	Y	Y	Y	Y	**P**	Y	Y	Y	Y	Y	Y	Y	Y	N	N	Y	Y	Y	N	Y	Y	Y	Y	Y	Y	Y	Y	24	Excellent
Buschmann et al. 2015	Y	Y	Y	Y	**Y**	Y	Y	Y	Y	Y	Y	/	Y	N	Y	Y	Y	Y	Y	Y	Y	Y	Y	Y	Y	Y	Y	26	Excellent
Cunningham et al. 2019	Y	Y	Y	Y	**N**	Y	Y	Y	Y	Y	/	/	Y	N	N	Y	Y	Y	Y	Y	/	/	N	N	N	Y	N	16	Fair
Cycyk et al. 2020	Y	Y	Y	Y	**P**	Y	Y	Y	Y	N	/	/	Y	N	N	Y	Y	Y	Y	Y	N	/	N	N	N	Y	N	16	Fair
Falkus et al. 2016	Y	Y	Y	Y	**N**	Y	Y	Y	Y	N	Y	Y	Y	N	Y	Y	Y	Y	Y	Y	/	/	N	N	N	Y	N	18	Fair
McDonald et al. 2019	Y	Y	Y	Y	**P**	Y	N	Y	Y	N	/	/	Y	N	N	Y	N	N	Y	Y	/	/	N	N	/	Y	N	13	Poor
McGowan et al. 2024	Y	Y	Y	Y	**Y**	Y	Y	Y	Y	Y	Y	/	N	N	Y	Y	Y	Y	Y	Y	Y	Y	Y	Y	Y	Y	Y	25	Excellent
Mettler et al. 2023	Y	Y	Y	Y	**P**	Y	Y	Y	Y	N	Y	/	Y	N	N	Y	Y	Y	Y	Y	/	N	N	N	N	Y	N	17	Fair
Moore et al. 2014	Y	Y	Y	Y	**P**	Y	Y	Y	Y	N	/	/	Y	N	N	Y	Y	Y	Y	Y	/	/	N	N	/	Y	N	16	Fair
Nielsen et al. 2023	Y	Y	Y	Y	**P**	Y	Y	Y	Y	N	Y	Y	Y	N	N	Y	N	Y	/	Y	Y	Y	Y	N	N	/	N	18	Fair
Olson et al. 2016	Y	Y	Y	Y	**P**	Y	Y	Y	Y	Y	Y	/	Y	N	N	Y	Y	Y	Y	Y	/	N	N	N	N	Y	N	18	Fair
Peredo et al. 2022	Y	Y	Y	Y	**P**	Y	Y	Y	Y	Y	Y	/	Y	N	Y	Y	Y	Y	Y	Y	Y	/	Y	Y	/	Y	Y	23	Good
Quinn et al. 2021	Y	Y	Y	Y	**P**	Y	Y	Y	Y	N	/	/	Y	N	N	Y	Y	Y	Y	Y	/	/	N	N	/	Y	N	16	Fair
Rajesh and Venkatesh 2019	Y	Y	Y	Y	**P**	Y	Y	Y	N	Y	/	/	Y	N	N	Y	Y	Y	Y	/	/	/	N	N	N	Y	N	15	Fair
Roberts et al. 2014	Y	Y	Y	Y	**P**	Y	N	Y	Y	N	/	/	Y	N	N	Y	Y	Y	Y	Y	/	/	N	/	N	Y	N	15	Fair
Roberts and Kaiser 2015	Y	Y	Y	Y	**Y**	Y	Y	Y	Y	Y	Y	/	Y	N	N	Y	Y	Y	Y	Y	Y	/	Y	Y	Y	Y	Y	24	Excellent
Sawyer et al. 2025	Y	Y	Y	Y	**P**	Y	Y	Y	N	Y	/	/	Y	N	/	Y	Y	Y	Y	Y	Y	Y	Y	/	/	/	Y	19	Good
Senent‐Capuz et al. 2021	Y	Y	Y	Y	**N**	Y	Y	Y	Y	Y	/	/	Y	N	N	Y	Y	Y	/	Y	Y	Y	N	N	N	/	N	16	Fair
Suttora et al. 2021	Y	Y	Y	Y	**P**	Y	Y	Y	Y	Y	/	/	Y	N	Y	Y	/	Y	Y	Y	Y	Y	N	N	Y	Y	N	20	Good
Tang et al. 2024	Y	Y	Y	Y	**P**	Y	Y	Y	Y	Y	/	/	Y	N	N	Y	Y	Y	Y	Y	N	N	N	N	/	Y	N	17	Fair
Vally et al. 2015	Y	Y	Y	Y	**P**	Y	Y	Y	Y	Y	Y	Y	Y	N	Y	Y	Y	Y	Y	Y	Y	Y	Y	Y	N	Y	N	24	Excellent
Vrinda and Kunnath 2023	Y	Y	Y	Y	**N**	Y	N	Y	Y	N	/	/	Y	N	N	Y	Y	Y	N	Y	/	/	N	N	/	Y	N	13	Poor
Wake et al. 2015	Y	Y	Y	Y	**P**	Y	Y	Y	Y	Y	Y	/	Y	N	Y	Y	Y	Y	Y	Y	Y	Y	Y	Y	Y	Y	Y	25	Excellent
Zuccarini et al. 2020	Y	Y	Y	Y	**N**	Y	Y	Y	Y	Y	/	/	/	N	/	Y	Y	Y	Y	Y	Y	Y	N	N	/	Y	N	17	Fair

Yes = Y (1 point), No = N (0 points), Unclear = / (0 points). Q5 only: Yes scores 2, Partially (‘P’) scores 1, No scores 0.

The nine RCTs generally exhibited higher methodological quality compared to the 17 non‐randomised studies. Among RCTs, six achieved Excellent ratings (Burgoyne et al. 2018; Buschmann et al. 2015; McGowan et al. 2024; Roberts and Kaiser 2015; Vally et al. 2015; Wake et al. 2015) and the remaining three were rated Good. None were rated Fair or Poor. Excellent‐rated RCTs demonstrated particular strengths in allocation concealment (Q24), adequate confounder adjustment (Q25), accounting for losses to follow‐up (Q26), performance of power analysis (Q27), and accurate reporting of *p*‐values (Q10). Excellent‐rated studies also consistently reported participant characteristics lost to follow‐up (Q9) and frequently achieved blinding of outcome assessors (Q15).

In contrast, non‐randomised studies achieved no Excellent and only one Good rating, with 14 rated Fair and two rated Poor. This is unsurprising given the diversity of research designs, including small‐scale, pilot and feasibility studies. Non‐randomised studies rated Good or Fair generally scored well on reporting quality, external validity, and statistical rigour but inevitably lost points on RCT‐specific criteria (Q23, 24) and frequently on confounding adjustment in analyses (Q25), unclear selection bias (Q11, 12), and lack of outcome assessor blinding (Q15). The two Poor‐rated studies exhibited weaknesses including limited reporting of random variability estimates (Q7), incomplete reporting of participant attrition (Q9), and insufficient adjustment for confounding variables (Q25).

Understandably, no included study was able to achieve participant blinding (Q14). There was also limited outcome assessor blinding across studies (Q15; achieved in only 27% overall), and a lack of power analyses (Q27; reported in only 31% of studies).

The more rigorously designed studies generally yielded more conservative effect size estimates, and those employing standardised language assessments produced more consistent findings than studies using informal or observational measures.

### 3.2 Narrative Synthesis

The primary outcome was formal or informal language assessment score, measured via caregiver‐reported observation checklists, researcher‐coded observation schedules, caregiver‐reported and researcher‐reported checklists related to the frequency and range of specified communication behaviours observed, and formal language assessment tools.

Secondary outcomes were caregiver‐reported confidence in supporting language development, and child educational attainment, well‐being, inclusion, and cognitive skills such as phonological working memory (which we considered to be language‐related outcomes).

Individual outcome measures used in included studies are reported in Table 2.

#### 3.2.1 Child Outcomes

##### 3.2.1.1 Language Skills

Six smaller‐scale non‐randomised studies found positive effects on child language measures. A case series by McDonald et al. (2019) (*N* = 7) measured the effectiveness of the Home Talk intervention on 26–31 month‐olds with primary expressive language delay, finding that 87% of children showed language use skills within the expected range for their chronological age following intervention. Another small‐scale case study by Mettler et al. (2023) (*N* = 5) found that all five children who took part in a caregiver‐implemented telehealth model of Vocabulary Acquisition and Usage for Late Talkers (VAULT) increased their age‐matched expressive vocabulary on formal language assessments. Moore et al. (2014) (*N* = 8) used the MacArthur–Bates Communicative Development Inventories (MBCDI) (Fenson et al. 2007) as an observational caregiver‐report language measure to explore the effect of Language and Play Every Day (LAPE) (Davies et al. 2016) on child vocabulary. This study also found that all children increased their age‐matched expressive vocabulary size. Senent‐Capuz et al. (2021) also used the MBCDI to measure the effect of It Takes Two to Talk—The Hanen Program for Parents (ITTT) (Hanen Centre 2016) on child language outcomes, with authors finding no observable intervention effect on children's expressive or receptive skills. Suttora et al. (2021) measured the effect of a caregiver‐implemented dialogic reading and focused stimulation intervention, finding that children in the intervention group demonstrated increased word types, word tokens and mean length of utterance although only the word tokens condition outcome was statistically significant. Although McDonald et al. (2019) did not report measures of intervention fidelity, unlike Moore et al. (2014), who documented these in detail, the inclusion of a clearly defined no‐treatment baseline phase in McDonald et al. offers a degree of empirical control when interpreting potential treatment effects. Conversely, in Moore et al., the same individuals conducted both assessment and intervention, which may introduce bias despite this study's stronger reporting of fidelity procedures.

A cohort study by Cunningham et al. (2019) measured the effect of Target Word, The Hanen Program for Parents of Children who are Late Talkers (Hanen Centre 2017) on children's scores on the MBCDI (Fenson et al. 2007) and the Focus on the Outcomes of Communication Under Six (FOCUS‐34) (Thomas‐Meyer et al. 2018). In this study, 75% of children receiving the intervention were judged to have made clinically meaningful change. Authors noted that it was not possible to directly attribute changes in communicative participation skills to the effect of the intervention due to the significant change in scores between the first two time points, both of which were before the start of the intervention. Another cohort study (Falkus et al. 2016) utilised measures of child's mean length of utterance (MLU) and the ratio of child to caregiver speech during recordings of naturalistic play after the provision of language support strategies, finding that both measures reached statistical significance (MLU *p* < 0.01; Ratio child to adult speech *p* < 0.001).

Sawyer et al. (2025) conducted a small‐scale RCT which indicated that the Parents Plus online intervention showed promising effects on children's vocabulary and morphosyntactic skills. Although the authors described their study as underpowered for statistical significance, as a small‐scale RCT it evidence offers stronger evidence of intervention effectiveness than the quasi‐experimental designs described above. The RCT conducted by Buschmann et al. 2015, *N* = 43) found that children who had received the Heidelberg Parent‐based Language Intervention (Buschmann et al. 2009) showed significantly higher scores than controls in the Expressive Vocabulary Test for 3 To 5 Year‐old Children (AWST‐R; Kiese‐Himmel 2005). It is worth noting that this study utilised an unusually long post‐test period of two years from baseline pretest. Another RCT by Bagner et al. (2016) found that a home‐based adaptation of Parent‐child Interaction Therapy used in a population of children at risk for language and behavioural problems had a significant positive effect on formal expressive language assessment scores (*N* = 60, *p* < 0.001, Cohen's *d* = 0.63). In a larger‐scale RCT (*N* = 208), Burgoyne et al. (2018) found that language scores in formal assessments probing expressive vocabulary and narrative skills were significantly higher for children receiving a caregiver‐mediated early language enrichment program than a motor skills control group at immediate post‐test, although this difference was reduced to non‐significant levels at 6‐month delayed post‐test. Another RCT by McGowan et al. (2024, *N* = 64) reported that at 24‐month follow‐up, a caregiver‐mediated early language enrichment program was associated with 80% decreased odds of a clinically significant language composite score on the Bayley Scales of Infant and Toddler Development (BSID)‐III (Bayley 2006).

###### 3.2.1.1.1 Narrative Skills

Burgoyne et al. (2018) reported that while a caregiver‐mediated early language enrichment program produced improvements in both language score (*d* = 0.21) and narrative skills (*d* = 0.36) immediately after the intervention, this effect size was only maintained on language at six‐month follow‐up (*d* = 0.34) and the effect size on children's narrative skills had declined (*d* = 0.08).

###### 3.2.1.1.2 Phonological Development and Speech Sound Inventory

One cohort study (Cunningham et al. 2019) reported a secondary outcome of consonant inventory, assessed via a speech sound checklist included within the Hanen program (McGowan et al. 2024). This study found a significant growth in consonant inventory following the Target Word intervention.

###### 3.2.1.1.3 Pre‐Literacy and Literacy Skills

Burgoyne et al. (2018) measured child outcomes related to early literacy skills. This RCT found that early language enrichment led to significantly increased letter‐sound knowledge and accuracy of regular word reading. Vrinda and Kunnath (2023) also reported a clear positive effect of intervention on emergent literacy skills as measured by the Get Ready to Read tool (GRTR) (Benmeleh 2011) with participants demonstrating improvement in their GRTR scores post‑intervention, regardless of baseline scores.

##### 3.2.1.2 Cognitive Development

A range of outcomes that could be described under the broad umbrella of cognitive development were reported in studies. Buschmann et al. (2015) measured aspects of phonological working memory as a secondary outcome, finding that the Heidelberg Parent‐Based Language Intervention produced a significant effect on phonological memory and episodic buffer assessed via non‐word repetition, word span, word order, and number recall tests at 2‐ year follow‐up. McGowan et al. (2024) found no significant difference in overall cognitive assessment performance in preterm children whose caregivers had taken part in a 6‐week language enrichment intervention in the neonatal intensive care unit, despite language assessment gains.

#### 3.2.2 Caregiver Outcomes

##### 3.2.2.1 Awareness of and Adherence to Strategies

Several smaller‐scale non‐randomised studies reported caregivers' adherence to language teaching strategies. Olson et al. (2016) measured caregivers' self‐ratings on a non‐standardised tool following the uTalk text messaging program. Following the intervention, there was a significant increase in caregivers reporting knowledge of language‐promoting activities for children (from 56% to 96%, *p* = 0.002). Caregivers also reported significantly higher engagement in language enrichment activities with their children, rising from 89% to 100% (*p* = 0.004). Rajesh and Venkatesh (2019) found a positive effect on caregiver adherence to language enrichment strategies taught during a caregiver‐mediated early language enrichment program, and on observational measures of child communication behaviours. Roberts (2014) found that three out of four caregivers who took part in intervention generalised their use of Enhanced Milieu Teaching (EMT) language support strategies to their everyday play in the home environment. Suttora et al. (2021) found that caregivers in their intervention group showed significant differences at 6‐month follow‐up in the use of taught language enrichment strategies, particularly functional caregiver speech features including more expansions of child utterances and more conversational talking while book reading.

In the randomised studies, Peredo et al. (2022) utilised observational measures of caregivers' adherence to language enrichment strategies during caregiver‐child interaction. Although results were underpowered for statistical significance, authors reported that they indicated the promise of an adapted Spanish language version of Enhanced Milieu Teaching, EMT en Español. The RCT by Roberts et al. (2014) (*N* = 95) found that caregivers in the EMT intervention group showed significant gains in their use of language facilitation strategies, for example the use of matched turns during interactions with their child.

#### 3.2.3 Relationships and Attachment

Nielsen et al. (2023) reported children's emotional and behavioural difficulties as well as caregivers’ relationship with their child via caregiver‐reported measures, describing a non‐significant but indicative trend toward improved outcomes in both areas following caregiver‐mediated spoken language intervention. This RCT (*N* = 102) used the Maternal Object Relations Scale (MORS) (Thomas‐Meyer et al. 2019), a caregiver‐report measure of attachment and caregiver/child relationships, and the Home Learning Environment Questionnaire (HLEQ) (Melvin et al. 2021), a caregiver‐report measure of the type and frequency of home activities predictive of children's later language skills, finding that the intervention group demonstrated consistently higher scores than controls on both measures.

#### 3.2.4 Factors Associated With Effectiveness

Evidence regarding the effect of baseline child factors was mixed, with one large study (Burgoyne et al. 2018) suggesting greatest benefits for children with lowest baseline scores, while Cycyk et al. (2020) found the opposite pattern in a smaller study. Notably, one study focusing on preterm infants (McGowan et al. 2024) demonstrated that early intervention in neonatal intensive care units significantly reduced the odds of clinically significant language delay at 24‐month follow‐up, suggesting potential benefits of preventative approaches for at‐risk populations.

There was an impact on intervention effectiveness associated with the qualifications of professionals delivering caregiver training programmes. Studies with interventions delivered by Speech and Language Therapists or highly trained specialists (e.g., Buschmann et al. 2009; Falkus et al. 2016; Roberts and Kaiser 2015) generally demonstrated larger effect sizes than those delivered by therapy assistants, research assistants or community members. However, an exception to this pattern is Vally et al. (2015), where trained community members effectively delivered a dialogic book‐sharing program with robust outcomes, while increasing its ecological validity to caregivers.

Face‐to‐face interventions were predominant (21 studies), while only five studies utilised remote or hybrid delivery (Akemoglu et al. 2022; Mettler et al. 2023; Olson et al. 2016; Quinn et al. 2021; Sawyer et al. 2025). The text message‐based intervention (Olson et al. 2016) demonstrated significant improvements in caregiver self‐efficacy. The telehealth interventions (Akemoglu et al. 2022; Mettler et al. 2023; Sawyer et al. 2025) showed promising language gains comparable to face‐to‐face interventions.

Positive outcomes were demonstrated by study authors who actively engaged with stakeholders to explore the social validity of interventions before embarking on full‐scale trials. For example, in participant groups recruited based on cultural or linguistic minority status (Cycyk et al. 2020; Olson et al. 2016), or socio‐economic deprivation (Burgoyne et al. 2018; McDonald et al. 2019; Vally et al. 2015), the success of interventions was linked to building rapport with communities, ensuring accessible session times and locations, and providing materials such as books and toys rather than expecting appropriate environmental resources to be available to families. Direct translation into a new language was not enough; content and strategies needed to take into account cultural differences, and families reported increased satisfaction when materials such as books reflected their traditions and values (Hammer et al. 2016; Peredo et al. 2022; Vally et al. 2015). With a client group experiencing low literacy and widely reporting no books in the home at baseline, Vally et al. (2015) found that a higher level of direct modelling and coaching of the strategies led to an increase in the success of the intervention and the acceptability of the advice provided to caregivers.

### 3.3 Meta‐Analysis

A table of the different language outcome measures utilised in each study, and which contributed to this pooled effect size in the three meta‐analyses, can be found in the Appendix. Standardised mean difference calculations were used to allow for the inclusion of studies with different effect sizes within forest plots (Polanin et al. 2016).

#### 3.3.1 Language Assessment Composite Score

**FIGURE 2:**
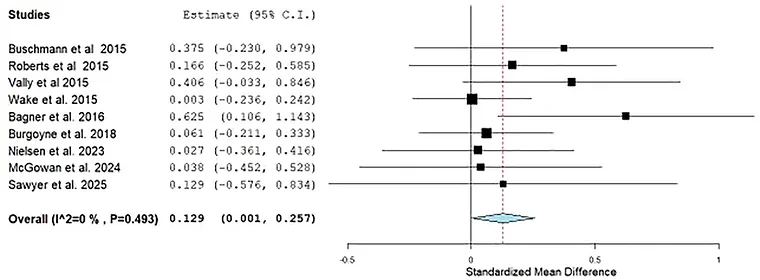
Forest plot of formal language assessment composite score for nine RCTs.

The forest plot in Figure 2 presents a meta‐analysis indicating the standardised mean difference for language composite scores across the included RCTs, with a total participant population of 997. Language composite outcomes included expressive and receptive language assessment scores measuring both structural language skills (e.g., grammar, morphology) and language content (e.g., expressive and receptive vocabulary). The random‐effects model revealed a small but statistically significant overall effect size, with a standardised mean difference of 0.129 (95% CI: 0.001 to 0.257, *p* = 0.048). This indicates a modest positive effect that just reaches statistical significance at the conventional threshold of 0.05. The analysis includes nine studies, with Wake et al. (2015) and Burgoyne et al. (2018) contributing the most weight (28.609% and 22.112%, respectively). The nine studies (df = 8) had low statistical heterogeneity, indicated by I^2^ of 0% and a non‐significant Cochran's Q‐test (*p* = 0.493), suggesting consistency in the observed effects across different studies and supporting the reliability of the pooled estimate. In this meta‐analysis, the modest positive effect size suggests a small but consistent improvement in language composite scores across the nine RCTs.

#### 3.3.2 Receptive Language Score

**FIGURE 3:**
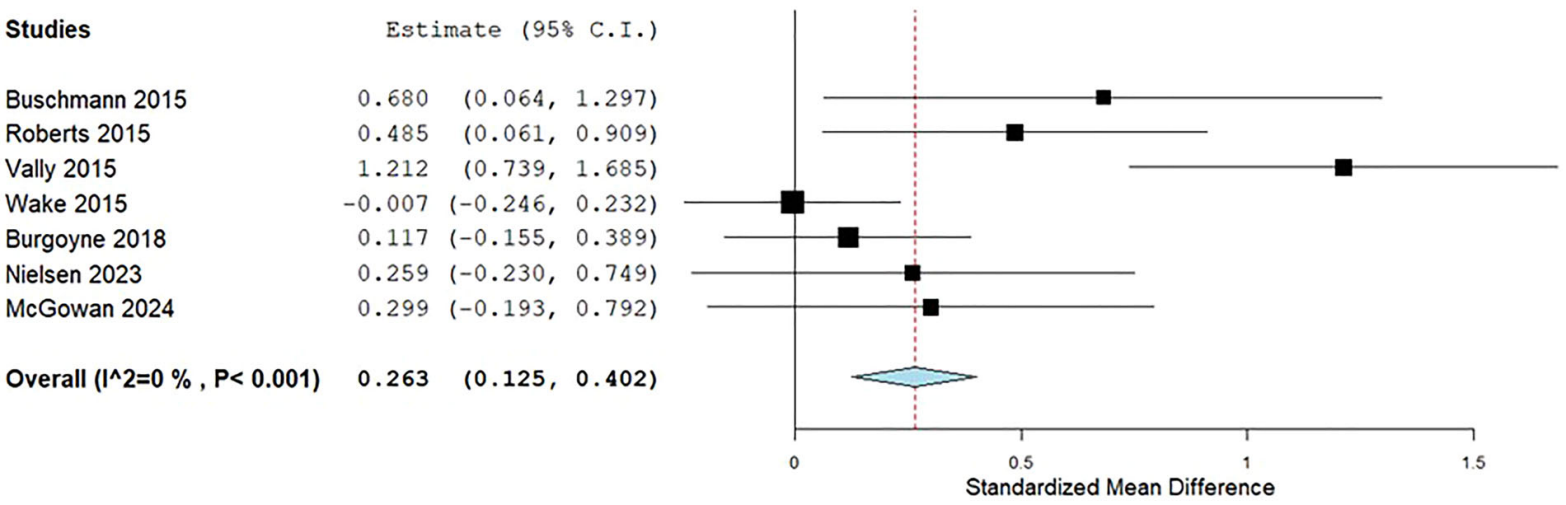
Forest plot of standardised mean difference for Receptive Language outcome for seven RCTs. Continuous Fixed‐Effect Model Inverse Variance used due to high heterogeneity.

The meta‐analysis of receptive language scores, which were reported by seven RCTs, seen in Figure 3, yielded a statistically significant overall effect size of SMD = 0.263 (95% CI [0.125, 0.402], *p* < 0.001). This represents a small but significant positive effect according to Cohen's conventions for interpreting standardised mean differences. Despite the use of an inverse variance model, significant heterogeneity was observed across the included studies (Q(6) = 24.314, *p* < 0.001), indicating substantial variability in effect sizes that cannot be attributed to sampling error alone. This heterogeneity suggests the presence of moderating variables that may influence the intervention effects on receptive language gains across different interventions, contexts, or populations.

#### 3.3.3 Expressive Language Score

**FIGURE 4:**
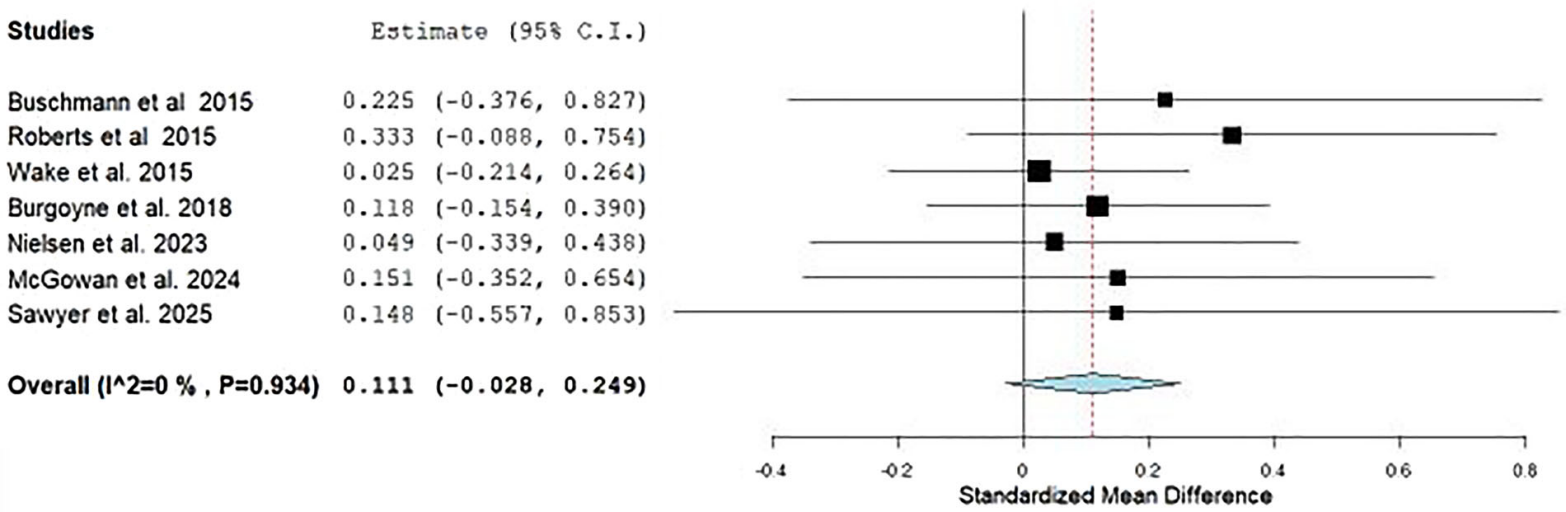
Forest plot of standardised mean difference for Expressive Language outcome for seven RCTs.

The forest plot for Expressive Language scores, which were reported by seven RCTs, is seen in Figure 4. The random‐effects model demonstrates a small positive effect that fails to reach statistical significance, with a standardised mean difference of 0.111 (95% CI: ‐0.028 to 0.249, *p* = 0.118). Heterogeneity estimates indicate consistency across studies (*I* ^2^ = 0, *Q* = 1.840, *p* = 0.934), suggesting that the studies are measuring the same underlying effect and that methodological or sampling differences had minimal impact on the results. Despite the homogeneity across studies, the overall effect remains inconclusive due to the non‐significant *p*‐value.

### 3.4 Summary of Results

This systematic review and meta‐analysis examined the effectiveness of caregiver‐mediated spoken language interventions for children under five at risk of Developmental Language Disorder. The methodological quality of included studies varied considerably. Higher‐quality studies typically reported more conservative effect sizes. Studies using standardised language assessments showed more consistent effects than those relying on informal or observational measures. This pattern underscores the importance of robust measurement in future research, ideally combining standardised assessments with ecologically valid observational measures to capture both language gains and functional improvements in communicative participation.

The meta‐analysis of nine Randomized Controlled Trials involving 947 children revealed significant positive effects of caregiver‐mediated interventions on overall and receptive language outcomes compared to control conditions. Three RCTs reported significant positive effects on formal language assessment scores, including expressive vocabulary and narrative skills (Bagner et al. 2016; Burgoyne et al. 2018; Buschmann et al. 2009), though Burgoyne et al. (2018) found that some benefits diminished at follow‐up assessment. McGowan et al. (2024) reported that caregiver‐mediated language intervention was associated with 80% decreased odds of clinically significant language delay at 24‐month follow‐up. Narrative synthesis identified further effectiveness patterns across child and caregiver outcomes.

Six smaller‐scale studies found positive effects on child language development but lacked sufficient power for statistical analysis, with interventions such as Home Talk (McDonald et al. 2019), VAULT (Mettler et al. 2023), and LAPE (Moore et al. 2014) showing improvements in children's expressive vocabulary and communication skills, while Senent‐Capuz et al. (2021) found no observable intervention effect and Suttora et al. (2021) reported non‐significant increases. Several cohort studies demonstrated statistically significant gains in children's word production, Mean Length of Utterance, and vocabulary scores following caregiver‐mediated interventions (Cunningham et al. 2019; Falkus et al. 2016; Suttora et al. 2021), although Cunningham et al. (2019) noted difficulties attributing changes solely to intervention effects. Caregiver‐mediated interventions positively influenced pre‐literacy skills, phonological development, and phonological working memory (Burgoyne et al. 2018; Buschmann et al. 2009; Cunningham et al. 2019).

Multiple studies found that caregivers successfully learned and implemented taught language enrichment strategies (Peredo et al. 2022; Rajesh and Venkatesh 2019; Roberts 2014; Suttora et al. 2021), with significant improvements in their use of language facilitation techniques during interactions with their children. One study reported increased caregiver self‐efficacy and awareness of language‐promoting activities (Olson et al. 2016).

## 4 Discussion

This systematic review set out to update and extend current evidence on the effectiveness of caregiver‐mediated spoken‐language interventions for children under five who show early indicators or risk factors associated with later Developmental Language Disorder (DLD). The first aim was to evaluate how these interventions influence children's language development, as well as broader aspects of child cognitive development, caregiver knowledge and confidence, and child‐caregiver relationships. The second aim was to explore the factors associated with greater intervention effectiveness. By synthesising recent research, the review sought to evaluate the strength, consistency, and limitations of the existing evidence base and identify implications for clinical practice and future research.

Caregiver‐mediated spoken language interventions generally showed positive effects on language outcomes in children under five across a range of small‐scale, non‐randomised studies. Evidence for broader child cognitive outcomes was mixed, with some gains in phonological memory but inconsistent effects on overall cognitive development. Caregiver outcomes were consistently positive, with studies reporting improved caregiver knowledge, confidence and use of strategies. There were also indications of stronger caregiver‐child relationships where these were measured. However, these findings were more variable and often underpowered for statistical significance. Several RCTs also demonstrated significant gains in children's expressive vocabulary, narrative skills, and early literacy. Meta‐analysis indicated a small but consistent positive effect of interventions on composite (expressive and receptive) language assessment scores, and on receptive language skills. Quantitative synthesis did not indicate a significant effect of intervention on expressive language skills. The review highlights the need for further high‐quality randomised trials to strengthen the evidence base and offer insight into the factors that contribute to more effective interventions.

### 4.1 Factors Influencing Intervention Effectiveness

Intervention effectiveness appeared to be influenced by several contextual factors. Programmes delivered by Speech and Language Therapists or highly trained specialists tended to have stronger effects, although there was some evidence that models of delivery by well‐trained community members could also be effective while offering increased ecological validity. Remote and telehealth approaches produced promising results comparable to face‐to‐face delivery. Finally, interventions that prioritised cultural relevance, stakeholder engagement, and accessible delivery models were more successful and acceptable to families, particularly those from socioeconomically or culturally marginalised groups.

Although follow‐up period duration varied by study design and was probably influenced by practical considerations such as funding, the range of follow‐up durations allowed some comparisons to be made. The studies reporting the most robust long‐term outcomes (Buschmann et al. 2015; McGowan et al. 2024) share certain characteristics: they involved higher dosage (multiple sessions over extended periods), included structured follow‐up support, and began intervention before 24 months. Conversely, studies with shorter intervention periods or limited follow‐up support (e.g., Burgoyne et al. 2018) showed diminishing effects over time. This pattern suggests that the sustainability of child language gains may depend on intervention intensity, duration, and the provision of maintenance strategies for caregivers. Longer study follow‐up periods could help stakeholders to understand whether positive language outcomes are maintained, and evaluate the holistic effect of early language intervention on attainment and wellbeing across the lifespan.

#### 4.1.1 Adaptation and Accessibility

Earlier reviews (Manz et al. 2010; Mol et al. 2008) found that the effectiveness of dialogic book reading interventions in supporting under‐fives’ language development was less effective for low‐income families, proposing that this was because the design and practices of the intervention lacked social validity outside the context of a white middle‐class sociocultural milieu. Recommendations included an examination of the cultural relevance and content of books supplied as part of the intervention. Authors also noted that families should be encouraged to employ the language with which they are most comfortable when engaging in interactive book reading, with due consideration to the value of bilingualism for child development. Our review offers additional evidence for the effectiveness of culturally‐adapted caregiver language intervention programmes in increasing ecological validity, caregiver self‐efficacy and engagement. Given that existing child language acquisition research draws overwhelmingly from English and other Indo‐European languages from the Global North (Kidd and Garcia 2022), the need for more generalisable theories of language acquisition may also limit understanding of effective intervention planning with families of diverse cultural and linguistic backgrounds.

### 4.2 Study Limitations

Given the prevalence and impact of DLD, there was a relatively small number of studies available to synthesise in this review, which may limit the generalisability of findings.

It should be noted that the heterogeneity estimates reported in this analysis may have limited reliability given the relatively small number of studies. I^2^ estimates can fluctuate significantly when fewer than 14 trials are included (Thorlund et al. 2012). Cochran's Q test may also be underpowered in this context, which can be ameliorated by adopting a lower *p*‐value threshold for appraising the statistical significance of heterogeneity estimates (Higgins 2008). Caution is therefore needed in interpreting the remarkably high homogeneity of effect found across the 7 RCTs that specifically reported expressive language outcomes.

### 4.3 Directions for Further Research

The heterogeneity observed in the receptive language meta‐analysis (Q(6) = 24.314, *p* < 0.001) suggests the presence of moderating variables. This may partly reflect methodological differences, particularly in outcome measurement approaches. Further investigation of the nature of these moderating factors would offer a better understanding of the conditions under which caregiver‐mediated preschool language interventions are the most effective for supporting the crucial domain of individuals’ language understanding. This is particularly important given the evidence that lower receptive skills are a predictor of poorer outcomes in DLD (Heidlage et al. 2020; O'Neill et al. 2019).

Conversely, although heterogeneity in this analysis was low, the finding regarding the lack of effectiveness of caregiver‐mediated early language intervention on building children's expressive skills is at odds with previous research. It may yet be the case that factors related to specific interventions and populations influence the effectiveness of caregiver‐mediated early language intervention on expressive skills. Further research is required into the causes of the low level of effectiveness found across the included studies, and the factors that may increase the effectiveness of caregiver‐mediated interventions on this domain of language development.

### 4.4 Clinical Implications

Two studies employed interventions that addressed both language and behavioural outcomes. Bagner et al. (2016) explored a home‐based adaptation of Parent‐child Interaction Therapy for children at risk for both language and behavioural problems, finding significant positive effects on expressive language (*p* < 0.001, Cohen's *d* = 0.63). Similarly, Nielsen et al. (2023) measured both language outcomes and emotional/behavioural difficulties, noting a trend toward improvements in both domains. These findings suggest potential synergistic effects of interventions designed to target both language and social‐emotional development. This may reflect the bidirectional relationship between language skills and emotional regulation (Hentges et al. 2021; Rautakoski et al. 2021). Future research that more explicitly examined these linked outcomes could support the development of caregiver‐mediated language interventions that address these interrelated developmental domains holistically.

Interventions’ effectiveness appeared to be influenced by the qualifications of those delivering the training to caregivers. This suggests that while specialist qualifications support effective implementation, carefully structured interventions with appropriate training protocols can also be successfully implemented by non‐specialists, potentially increasing scalability and cost‐effectiveness. Future studies systematically assessing the relationship between implementer qualifications, intervention fidelity and outcomes could help to optimise the allocation of resources within service delivery models.

The format of intervention delivery, such as face‐to‐face, hybrid, online or via text messaging, did not appear to be linked with effectiveness, indicating that a range of delivery modes offer potential for effective caregiver‐mediated language intervention. Future research could directly compare delivery modes using equivalent intervention content to matched groups, in order to determine optimal approaches for different populations and contexts. Studies that looked at the effect of remote (Akemoglu et al. 2022; Mettler et al. 2023; Sawyer et al. 2025) or hybrid delivery formats (Quinn et al. 2021) also offer insights into balancing service pressures, time constraints and service budgets with developing maximally efficient resource provision and increasing accessibility for families. In particular, Olson et al. (2016) explored the effectiveness of text messaging as a low‐intensity, accessible and highly cost‐effective means of engagement with culturally and linguistically diverse participants with low socioeconomic status. While this study's outcomes were self‐reported caregiver self‐efficacy and the social validity of the intervention, with no objective child language outcomes, results were encouraging regarding the acceptability, accessibility and cost‐effectiveness of text messaging as a targeted early caregiver‐mediated language intervention.

Similarly, Bagner et al. (2016) discuss the development of interventions that start earlier and take less time to complete, as a response to the high dropout rate seen in families under socioeconomic stress, with the authors linking this particularly to participants’ linguistic or cultural minority status in their own study. These areas of research indicate promising results for responsive care planning that takes into account the access needs of a wider range of families, such as those experiencing socioeconomic deprivation. These insights may prove helpful in developing provision for families experiencing the intersectional effects of heritable, widespread and under‐diagnosed DLD with other factors associated with lower access to and engagement with support services.

Although many studies had an element of cultural or sociolinguistic adaptations aimed at meeting the needs of a particular subset of the community or underserved population, no studies specifically mentioned adapting caregiver‐mediated language interventions to caregivers who may themselves have a language or communication difficulty. Due to the low diagnostic rate for DLD (Calder et al. 2022), these difficulties may be covert, masked and/or unknown both to participants and to the professionals working with them, and so authors who reported promising results of adapted interventions aimed at accessibility for caregivers living in areas of socioeconomic deprivation may have inadvertently included aspects of adaptation that mitigated access barriers associated with lower language skills. For example, Vally et al. (2015) offer insight into the effective adaptation of a book‐sharing intervention to a population typified by lower literacy, where study authors employed trained community members to deliver the intervention, increasing its cultural specificity and ecological validity. It may be useful to consider the application of some of the insights generated by these authors in designing interventions that better meet the needs of populations affected by intergenerational language difficulties.

More information is needed on the demographic characteristics of participants in order to better understand the effect of variables such as socioeconomic deprivation, adverse childhood experiences and different family and sociocultural support structures on the effectiveness of therapy provision, particularly when this relies so heavily upon the ability of families to be the main agents of environmental language intervention for their children. Particularly notable in the studies included in this review was the lack of investigation and/or reporting of the language skills available to caregivers in supporting their children's developmental needs. Caregiver language levels were not reported within any of the studies reviewed. We consider that this is a particularly important factor to consider in designing effective and accessible caregiver‐mediated early language interventions.

#### 4.4.1 Neuroaffirming Lens

Recently, a number of authors have challenged deficit‐based approaches to DLD, in line with broader shifts in disability research methodologies, which are increasingly informed by the social model of disability (U.P.I.A.S. 1976), and the neurodiversity paradigm (Singer 1998). For example, Lamoureux et al. (2024) argue against viewing language differences as inherently pathological, rather than as part of legitimate neurodevelopmental variation. Historical approaches to DLD research may overemphasise the normalisation of individuals’ language profiles, rather than the mitigation of environmental barriers to communication access (Hobson et al. 2024). There is evidence of a shift to reflect this in recent research designs. For example, when investigating the impact of DLD on peer interactions, Cullen et al. (2024) explicitly focused on the self‐reported experiences of children with DLD. Participatory methodologies that include individuals with DLD in research design are an emerging priority within the evidence base. Aussems et al. (2024) reported the involvement of adolescents with DLD as co‐researchers, generating novel insight into their lived experience of communication differences. Authors (e.g., Chadd et al. 2020; Janik Blaskova and Gibson 2021; Larson 2025) advocate for greater inclusion of individuals with DLD and other stakeholders throughout the research process, with benefits including developing more relevant outcome measures and uncovering previously overlooked priorities such as promoting social participation and identity development for people with DLD. Adoption of a neuroaffirming lens, and inclusion of individuals with DLD in research would allow outcome measures that more accurately reflect the lived experiences and priorities of affected people.

## 5 Conclusion

Overall, the literature on caregiver‐mediated spoken language interventions for children under five presenting with markers for DLD shows evidence for a positive effect on children's language outcomes, particularly on receptive language, and indicative evidence of wider child and caregiver benefits. Crucially, our review offers robust evidence that early family‐based environmental language enrichment interventions stand to have a significant effect on ameliorating the risk factors and early markers for DLD experienced by 12% of preschool children. These insights can inform service planning by healthcare services such as Speech and Language Therapy, and universal and targeted perinatal and early years family support. More robust evidence is required regarding the most effective format and design of these interventions to maximise accessibility and social validity for all caregivers, for example in families impacted by the same factors which may impart additional risk for early language development concerns in their children, such as socioeconomic deprivation. There is a particular lack of evidence of the factors which impact the effectiveness of interventions for caregivers who experience features of DLD. In order to drive positive change in this area individual and public awareness, professional knowledge and diagnostic rates of DLD must be raised.

## Conflicts of Interest

The authors declare no conflicts of interest.

## Supporting information




**Supporting Information**: jlcd70304‐supp‐0001‐SuppMat.docx

**Supporting Information**: jlcd70304‐supp‐0002‐SuppMat.docx

**Supporting Information**: jlcd70304‐supp‐0003‐SuppMat.docx

**Supporting Information**: jlcd70304‐supp‐0004‐SuppMat.docx

**Supporting Information**: jlcd70304‐supp‐0005‐SuppMat.docx

## Data Availability

The data that support the findings of this study are available from the corresponding author upon reasonable request.

## Appendix 1

### Modified Downs and Black Methodological Quality Checklist

#### List of Questions

1. Is the hypothesis/aim/objective of the study clearly described?
2. Are the main outcomes to be measured clearly described in the Introduction or Methods section?
3. Are the characteristics of the patients included in the study clearly described?
4. Are the interventions of interest clearly described?
5. *Are the distributions of principal confounders in each group of subjects to be compared clearly described? (Yes/No/Partially – 2 points maximum)*
6. Are the main findings of the study clearly described?
7. Does the study provide estimates of the random variability in the data for the main outcomes?
8. Have all important adverse events that may be a consequence of the intervention been reported?
9. Have the characteristics of patients lost to follow‐up been described?
10. Have actual probability values been reported (e.g., 0.035 rather than <0.05) for the main outcomes except where the probability value is less than 0.001?
11. Were the subjects asked to participate in the study representative of the entire population from which they were recruited?
12. Were those subjects who were prepared to participate representative of the entire population from which they were recruited?
13. Were the staff, places, and facilities where the patients were treated, representative of the treatment the majority of patients receive?
14. Was an attempt made to blind study subjects to the intervention they have received?
15. Was an attempt made to blind those measuring the main outcomes of the intervention?
16. If any of the results of the study were based on “data dredging”, was this made clear?
17. In trials and cohort studies, do the analyses adjust for different lengths of follow‐up of patients, or in case‐control studies, is the time period between the intervention and outcome the same for cases and controls?
18. Were the statistical tests used to assess the main outcomes appropriate?
19. Was compliance with the intervention/s reliable?
20. Were the main outcome measures used accurate (valid and reliable)?
21. Were the patients in different intervention groups (trials and cohort studies) or were the cases and controls (case‐control studies) recruited from the same population?
22. Were study subjects in different intervention groups (trials and cohort studies) or were the cases and controls (case‐control studies) recruited over the same period of time?
23. Were study subjects randomized to intervention groups?
24. Was the randomized intervention assignment concealed from both patients and health care staff until recruitment was complete and irrevocable?
25. Was there adequate adjustment for confounding in the analyses from which the main findings were drawn?
26. Were losses of patients to follow‐up taken into account?
27. Was a power analysis performed?

**Table:**

Score	Rating
0–14	Poor
15–18	Fair
19–23	Good
24–28	Excellent

Yes = 1, No/Unclear = 0, Total score 28.

## References

[jlcd70304-bib-0001] Adamson, L. B. , A. P. Kaiser , C. S. Tamis‐LaMonda , M. T. Owen , and N. Dimitrova . 2020. “The Developmental Landscape of Early Parent‐Focused Language Intervention.” Early Childhood Research Quarterly 50: 59–67. 10.1016/j.ecresq.2018.11.005.

[jlcd70304-bib-0002] Akemoglu, Y. , V. Hinton , D. Laroue , and V. Jefferson . 2022. “A Parent‐Implemented Shared Reading Intervention via Telepractice.” Journal of Early Intervention 44, no. 2: 190–210.

[jlcd70304-bib-0003] Allen, A. , and S. Spencer . 2022. “Regimes of Motherhood: Social Class, the Word Gap and the Optimisation of Mothers' Talk.” The Sociological Review 70, no. 6: 1181–1198. 10.1177/00380261221104378.

[jlcd70304-bib-0004] American Psychiatric Association . 2013. Diagnostic and Statistical Manual of Mental Disorders, 5th ed. American Psychiatric Association. 10.1176/appi.books.9780890425596.

[jlcd70304-bib-0005] Aussems, K. , J. Isarin , A. Niemeijer , and C. Dedding . 2024. “Working Together as Scientific and Experiential Experts: How Do Current Ethical PAR‐Principles Work in a Research Team With Young Adults With Developmental Language Disorder?” Educational Action Research 32, no. 2: 311–326. 10.1080/09650792.2022.2130386. 38504698 PMC10946222

[jlcd70304-bib-0006] Bagner, D. M. , D. Garcia ,, and R. Hill . 2016. “Direct and Indirect Effects of Behavioral Parent Training on Infant Language Production.” Behavior Therapy 47, no. 2: 184–197. 10.1016/j.beth.2015.11.001. 26956651 PMC4876808

[jlcd70304-bib-0007] Bayley, N. 2006. Bayley Scales of Infant and Toddler Development, 3rd ed. Harcourt Assessment.

[jlcd70304-bib-0008] Beitchman, J. H. , R. Nair , M. Clegg , B. Ferguson , and P. Patel . 1986. “Prevalence of Psychiatric Disorders in Children With Speech and Language Disorders.” Journal of the American Academy of Child Psychiatry 25, no. 4: 528–535. 3489024 10.1016/s0002-7138(10)60013-1

[jlcd70304-bib-0009] Benmeleh, E. 2011. Get Ready to Read!: Assessing the Efficacy of an Emergent Literacy Screening Tool. Nova Southeastern University.

[jlcd70304-bib-0010] Bishop, D. V. M. , B. Clark , G. Conti‐Ramsden , C. F. Norbury , and M. J. Snowling . 2012. “RALLI: An Internet Campaign for Raising Awareness of Language Learning Impairments.” Child Language Teaching and Therapy 28, no. 3: 259–262.

[jlcd70304-bib-0011] Bishop, D. V. M. , M. J. Snowling , P. A. Thompson , T. Greenhalgh , and Catalise Consortium . 2016. “CATALISE: A Multinational and Multidisciplinary Delphi Consensus Study. Identifying Language Impairments in Children.” PLoS ONE 11, no. 7: e0158753. 10.1371/journal.pone.0158753. 27392128 PMC4938414

[jlcd70304-bib-0012] Bishop, D. V. M. , M. J. Snowling , P. A. Thompson , et al. 2017. “Phase 2 of CATALISE: A Multinational and Multidisciplinary Delphi Consensus Study of Problems With Language Development: Terminology.” Journal of Child Psychology and Psychiatry 58, no. 10: 1068–1080. 10.1111/jcpp.12721. 28369935 PMC5638113

[jlcd70304-bib-0013] Brownell, R. 2000. Expressive One‐word Picture Vocabulary Test: EOWPVT. Academic Therapy Publications.

[jlcd70304-bib-0014] Burgoyne, K. , R. Gardner , H. Whiteley , M. J. Snowling , and C. Hulme . 2018. “Evaluation of a Parent‐Delivered Early Language Enrichment Programme: Evidence From a Randomised Controlled Trial.” Journal of Child Psychology and Psychiatry 59, no. 5: 545–555. 28940192 10.1111/jcpp.12819

[jlcd70304-bib-0015] Buschmann, A. , B. Jooss , A. Rupp , F. Feldhusen , J. Pietz , and H. Philippi . 2009. “Parent Based Language Intervention for 2‐Year‐Old Children With Specific Expressive Language Delay: A Randomised Controlled Trial.” Archives of Disease in Childhood 94, no. 2: 110–116. 18703544 10.1136/adc.2008.141572 PMC2614563

[jlcd70304-bib-0016] Buschmann, A. , B. Multhauf , M. Hasselhorn , and J. Pietz . 2015. “Long‐Term Effects of a Parent‐Based Language Intervention on Language Outcomes and Working Memory for Late‐Talking Toddlers.” Journal of Early Intervention 37, no. 3: 175–189. 10.1177/1053815115609384.

[jlcd70304-bib-0017] Calder, S. D. , C. G. Brennan‐Jones , M. Robinson , A. Whitehouse , and E. Hill . 2022. “The Prevalence of and Potential Risk Factors for Developmental Language Disorder at 10 Years in the Raine Study.” Journal of Paediatrics and Child Health 58, no. 11: 2044–2050. 35922883 10.1111/jpc.16149 PMC9804624

[jlcd70304-bib-0018] Caselli, M. C. , A. Bello , P. Rinaldi , S. Stefanini , and P. Pasqualetti . 2015. “Il primo vocabolario del bambino: Gesti, Parole e Frasi.” Valori Di Riferimento Fra 8: 8–36.

[jlcd70304-bib-0020] Chadd, K. E. , A. A. Kulkarni , and L. M. Longhurst . 2020. “Involving Individuals With Developmental Language Disorder and Their Parents/Carers in Research Priority Setting.” Journal of Visualized Experiments 6, no. 160: 61267. 10.3791/61267. 32568234

[jlcd70304-bib-0021] Chandler, J. , M. Cumpston , T. Li , M. J. Page , and V. Welch . 2019. “Cochrane Handbook for Systematic Reviews of Interventions.” Hoboken: Wiley 4, no. 1002: 14651858.

[jlcd70304-bib-0201] Chang, Y. E. 2017. “Pathways From Mothers' early Social Support to Children's Language Development at Age 3.” *Infant & Child Development*. 26, no. 6.

[jlcd70304-bib-0022] Conti‐Ramsden, G. , K. Durkin , U. Toseeb , N. Botting , and A. Pickles . 2018. “Education and Employment Outcomes of Young Adults With a History of Developmental Language Disorder.” International Journal of Language & Communication Disorders 53, no. 2: 237–255. 29139196 10.1111/1460-6984.12338 PMC5873379

[jlcd70304-bib-0023] Cullen, H. , S. Billingham , and M. C. St Clair . 2024. “How Do Children With Language Disorder Perceive Their Peer Interactions? A Qualitative Investigation.” Autism & Developmental Language Impairments 9: 23969415241275775. 10.1177/23969415241275775. 39221432 PMC11365031

[jlcd70304-bib-0024] Cunningham, B. J. , E. Kwok , C. Earle , and J. O. Cardy . 2019. “Exploring Participation and Impairment‐Based Outcomes for Target Word: A Parent‐Implemented Intervention for Preschoolers Identified as Late‐to‐Talk.” Child Language Teaching & Therapy 35, no. 2: 145–164. 10.1177/0265659019846931.

[jlcd70304-bib-0025] Cycyk, L. M. , H. W. Moore , and S. De Anda . 2020. “Adaptation of a Caregiver‐Implemented Naturalistic Communication Intervention for Spanish‐Speaking Families of Mexican Immigrant Descent: A Promising Start.” American Journal of Speech‐Language Pathology 29, no. 3: 1260–1282. 10.1044/2020_AJSLP-19-00142. 32750276

[jlcd70304-bib-0026] Davies, J. , S. Schofield , and J. Sigafoos . 2016. “Language and Play every Day (LAPE): A Communication Development Program for Young Children.” Special Education Perspectives 25, no. 1: 36–53.

[jlcd70304-bib-0027] Dockrell, J. E. , C. L. Forrest , J. Law , S. Mathers , and J. Charlton . 2022. “Screening for Language Difficulties in Disadvantaged Populations on Entry to Early Years Education: Challenges and Opportunities.” Frontiers in Pediatrics 10: 833603. 35601421 10.3389/fped.2022.833603 PMC9119430

[jlcd70304-bib-0028] Downs, S. H. , and N. Black . 1998. “The Feasibility of Creating a Checklist for the Assessment of the Methodological Quality Both of Randomised and Non‐Randomised Studies of Health Care Interventions.” Journal of Epidemiology & Community Health 52, no. 6: 377–384. 9764259 10.1136/jech.52.6.377 PMC1756728

[jlcd70304-bib-0029] Dubois, P. , M. C. St‐Pierre , C. Desmarais , and F. Guay . 2020. “Young Adults With Developmental Language Disorder: A Systematic Review of Education, Employment, and Independent Living Outcomes.” Journal of Speech, Language, and Hearing Research 63, no. 11: 3786–3800. 10.1044/2020_JSLHR-20-00127 33022192

[jlcd70304-bib-0030] Dunn, L. , L. Dunn , B. Styles , and J. Sewell . 2009. British Picture Vocabulary Scale (BPVS‐III), 3rd ed. *National Foundation for Educational Research* .

[jlcd70304-bib-0031] Dunn, L. M. , and L. M. Dunn . 2007. Peabody Picture Vocabulary Test, 4th ed. *PsycTESTS Dataset* .

[jlcd70304-bib-0032] Eadie, P. , L. Conway , B. Hallenstein , F. Mensah , C. McKean , and S. Reilly . 2018. “Quality of Life in Children With Developmental Language Disorder.” International Journal of Language & Communication Disorders 53: 799–810. 10.1111/1460-6984.12385. 29577529

[jlcd70304-bib-0033] Edwards, S. , M. Garman , A. Hughes , C. Letts , and I. Sinka . 1999. “Assessing the Comprehension and Production of Language in Young Children: An Account of the Reynell Developmental Language Scales III.” International Journal of Language & Communication Disorders 34, no. 2: 151–171. 15587011 10.1080/136828299247487

[jlcd70304-bib-0034] Falkus, G. , C. Tilley , C. Thomas , et al. 2016. “Assessing the Effectiveness of Parent–Child Interaction Therapy With Language Delayed Children: A Clinical Investigation.” Child Language Teaching and Therapy 32, no. 1: 7–17.

[jlcd70304-bib-0035] Fenson, L. , V. A. Marchman , D. J. Thal , P. S. Dale , J. S. Reznick , and E. Bates . 2006. MacArthur‐Bates Communicative Development Inventories. PsycTESTS Dataset.

[jlcd70304-bib-0036] Fenson, L. , V. A. Marchman , D. J. Thal , P. S. Dale , J. S. Reznick , and E. Bates . 2007. MacArthur‐Bates Communicative Development Inventories. 2nd ed. PsycTESTS Dataset.

[jlcd70304-bib-0037] Gibbard, D. , and C. Smith . 2016. “A Transagency Approach to Enabling Access to Parent‐Based Intervention for Language Delay in Areas of Social Disadvantage: A Service Evaluation.” Child Language Teaching & Therapy 32, no. 1: 19–33. 10.1177/0265659014567785.

[jlcd70304-bib-0202] Goh, S. K. , S. Griffiths , and C. F. Norbury . 2021. “Sources of Variability in the Prospective Relation of Language to Social, Emotional, and Behavior Problem Symptoms: Implications for Developmental Language Disorder.” Journal of Abnormal Psychology 130, no. 6: 676. 34553962 10.1037/abn0000691 PMC8459610

[jlcd70304-bib-0038] Goldin‐Meadow, S. , S. C. Levine , L. V. Hedges , J. Huttenlocher , S. W. Raudenbush , and S. L. Small . 2014. “New Evidence About Language and Cognitive Development Based on a Longitudinal Study: Hypotheses for Intervention.” American Psychologist 69, no. 6: 588. 24911049 10.1037/a0036886 PMC4159405

[jlcd70304-bib-0039] Greenland, S. , and K. O'Rourke . 2012. “Meta‐Analysis.” In Modern Epidemiology, edited by K. J. Rothman 3rd ed., 652–682. Lippincott Williams & Wilkins.

[jlcd70304-bib-0040] Grimm, H. 2001. Sprachentwicklungstest für Drei‐bis Fünfjährige Kinder: SETK 3‐5; Diagnose von Sprachverarbeitungsfähigkeiten und Auditiven Gedächtnisleistungen. Hogrefe, Verlag für Psychologie.

[jlcd70304-bib-0041] Hamilton, A. , K. Plunkett , and G. Schafer . 2000. “Infant Vocabulary Development Assessed With a British Communicative Development Inventory.” Journal of Child Language 27, no. 3: 689–705. 11089344 10.1017/s0305000900004414

[jlcd70304-bib-0042] Hammer, C. S. , and B. Sawyer . 2016. “Effects of a Culturally Responsive Interactive Book‐Reading Intervention on the Language Abilities of Preschool Dual Language Learners: A Pilot Study.” HS Dialog: The Research to Practice Journal for the Early Childhood Field 19, no. 2: 59–79.

[jlcd70304-bib-0043] Hanen Centre . 2016. It Takes Two to Talk: The Hanen Program for Parents of Children With Language Delays. Hanen Centre.

[jlcd70304-bib-0019] Hanen Centre . 2017. Target Word: The Hanen Program for Parents of Children Who Are Late Talkers. Hanen Centre.

[jlcd70304-bib-0044] Hart, B. , T. R. Risley , and J. R. Kirby . 1997. “Meaningful Differences in the Everyday Experience of Young American Children.” Canadian Journal of Education 22, no. 3: 323.

[jlcd70304-bib-0045] Heidlage, J. K. , J. E. Cunningham , A. P. Kaiser , et al. 2020. “The Effects of Parent‐Implemented Language Interventions on Child Linguistic Outcomes: A Meta‐Analysis.” Early Childhood Research Quarterly 50, no. Pt. 1: 6–23. 10.1016/j.ecresq.2018.12.006.

[jlcd70304-bib-0046] Hentges, R. F. , C. Devereux , S. A. Graham , and S. Madigan . 2021. “Child Language Difficulties and Internalizing and Externalizing Symptoms: A Meta‐Analysis.” Child Development 92, no. 4: e691–e715. 33491805 10.1111/cdev.13540

[jlcd70304-bib-0047] Hidecker, M. J. C. , B. J. Cunningham , N. Thomas‐Stonell , B. Oddson , and P. Rosenbaum . 2017. “Validity of the Communication Function Classification System for Use With Preschool Children With Communication Disorders.” Developmental Medicine & Child Neurology 59, no. 5: 526–530. 28084630 10.1111/dmcn.13373

[jlcd70304-bib-0048] Higgins, J. 2008. Cochrane Handbook for Systematic Reviews of Interventions Version 5.0. 1. The Cochrane Collaboration.

[jlcd70304-bib-0049] Higgins, J. P. T. , and S. G. Thompson . 2002. “Quantifying Heterogeneity in a Meta‐Analysis.” Statistics in Medicine 21, no. 11: 1539–1558. 10.1002/sim.1186. 12111919

[jlcd70304-bib-0050] Hobson, H. M. , U. Toseeb , and J. L. Gibson . 2024. “Developmental Language Disorder and Neurodiversity: Surfacing Contradictions, Tensions and Unanswered Questions.” International Journal of Language & Communication Disorders 59, no. 4: 1505–1516. 38275081 10.1111/1460-6984.13009

[jlcd70304-bib-0051] Hoyne, C. , and S. M. Egan . 2022. “ABCs and 123s: A Large Birth Cohort Study Examining the Role of the Home Learning Environment in Early Cognitive Development.” Journal of Experimental Child Psychology 221: 105424. 35447427 10.1016/j.jecp.2022.105424

[jlcd70304-bib-0052] Jackson‐Maldonado, D. , D. Thal , L. Fenson , V. Marchman , T. Newton , and B. Conboy . 2021. MacArthur Inventarios Del Desarrollo de Habilidades Comunicativas (Inventarios): User's Guide and Technical Manual. Brookes Publishing.

[jlcd70304-bib-0053] Janik Blaskova, L. , and J. L. Gibson . 2021. “Reviewing the Link Between Language Abilities and Peer Relations in Children With Developmental Language Disorder: The Importance of Children's Own Perspectives.” Autism & Developmental Language Impairments 6: 23969415211021515. 10.1177/23969415211021515. 36381523 PMC9620691

[jlcd70304-bib-0054] Kaufman, A. S. , and N. L. Kaufman . 1983. Kaufman Assessment Battery for Children. *APA PsycTests* . 10.1037/t27677-000.

[jlcd70304-bib-0055] Kidd, E. , and R. Garcia . 2022. “How Diverse Is Child Language Acquisition Research?” First Language 42, no. 6: 703–735. 10.1177/01427237221100138 PMC9605524 36310838

[jlcd70304-bib-0056] Kiese‐Himmel, C. 2005. AWST‐R‐Aktiver Wortschatztest für 3‐bis 5‐jährige Kinder. Beltz Test Göttingen.

[jlcd70304-bib-0058] Kim, J. H. , B. Davies , and N. Xu Rattanasone . 2023. “Have You Heard of Developmental Language Disorder? An Online Survey.” Communication Disorders Quarterly 44, no. 4: 228–238.

[jlcd70304-bib-0059] Lakkanna, S. , K. Venkatesh ,, and J. Bhat . 2008. Assessment of Language Development. Omni Therapy Services.

[jlcd70304-bib-0060] Lamoureux, G. , A. Tessier , S. Finlay , and I. Verduyckt . 2024. “Critical Perspectives in Speech‐Language Therapy: Towards Inclusive and Empowering Language Practices.” Disabilities 4, no. 4: 1006–1018. 10.3390/disabilities4040062.

[jlcd70304-bib-0061] Lanjekar, P. D. , S. H. Joshi , P. D. Lanjekar , and V. Vasant . 2022. “The Effect of Parenting and the Parent‐Child Relationship on a Child's Cognitive Development: A Literature Review.” Cureus 14, no. 10: e30574. 36420245 10.7759/cureus.30574 PMC9678477

[jlcd70304-bib-0062] Larson, C. 2025. “Exploring Self‐Reported Quality of Life in Developmental Language Disorder.” Perspectives of the ASHA Special Interest Groups 10, no. 1: 136–148. 10.1044/2024_PERSP-24-00122. 41098905 PMC12520234

[jlcd70304-bib-0063] Law, J. , J. Charlton , J. Dockrell , M. Gascoigne , C. McKean , and A. Theakston . 2017. Early Language Development: Needs, Provision, and Intervention for Preschool Children From Socio‐economically Disadvantage Backgrounds. Education Endowment Foundation.

[jlcd70304-bib-0064] Law, J. , S. Reilly ,, and C. McKean . 2022. Language Development: Individual Differences in a Social Context. Cambridge University Press.

[jlcd70304-bib-0065] Le, H. N. , F. Mensah , P. Eadie , et al. 2021. “Health‐Related Quality of Life of Children With Low Language From Early Childhood to Adolescence: Results From an Australian Longitudinal Population‐Based Study.” Journal of Child Psychology and Psychiatry 62, no. 3: 349–356. 32488955 10.1111/jcpp.13277

[jlcd70304-bib-0066] MacWhinney, B. 2000. The CHILDES Project: Tools for Analyzing Talk: Volume I: Transcription Format and Programs, Volume II: The Database. MIT Press One Rogers Street.

[jlcd70304-bib-0067] Manz, P. H. , C. Hughes , E. Barnabas , C. Bracaliello , and M. Ginsburg‐Block . 2010. “A Descriptive Review and Meta‐Analysis of Family‐Based Emergent Literacy Interventions: To What Extent Is the Research Applicable to Low‐Income, Ethnic‐Minority or Linguistically‐Diverse Young Children?” Early Childhood Research Quarterly 25, no. 4: 409–431.

[jlcd70304-bib-0068] Mariscal, S. , S. López‐Ornat , C. Gallego , P. Gallo , A. Karousou , and M. Martínez . 2007. “La Evaluación del Desarrollo Comunicativo y Lingüístico Mediantela Versión Española de los Inventarios MacArthur‐Bates.” Psicothema 19, no. 2: 190–197. 17425886

[jlcd70304-bib-0069] Masek, L. R. , S. J. Paterson , R. M. Golinkoff , et al. 2021. “Beyond Talk: Contributions of Quantity and Quality of Communication to Language Success Across Socioeconomic Strata.” Infancy 26, no. 1: 123–147. 33306866 10.1111/infa.12378

[jlcd70304-bib-0070] McDonald, D. , S. Colmer , S. Guest , D. Humber , C. Ward , and J. Young . 2019. “Parent‐Implemented Language Intervention Delivered by Therapy Assistants for Two‐Year‐Olds at Risk of Language Difficulties: A Case Series.” Child Language Teaching & Therapy 35, no. 2: 113–124. 10.1177/0265659019842244.

[jlcd70304-bib-0071] McGowan, E. C. , M. Caskey , R. Tucker , and B. R. Vohr . 2024. “A Randomized Controlled Trial of a Neonatal Intensive Care Unit Language Intervention for Parents of Preterm Infants and 2‐Year Language Outcomes.” The Journal of Pediatrics 264: 113740. 10.1016/j.jpeds.2023.113740. 37717908

[jlcd70304-bib-0072] McKean, C. , and S. Reilly . 2023. “Creating the Conditions for Robust Early Language Development for All: Part Two: Evidence Informed Public Health Framework for Child Language in the Early Years.” International Journal of Language and Communication Disorders 58: 2242–2264. 10.1111/1460-6984.12927. 37431980

[jlcd70304-bib-0203] McKean, C. , S. Reilly , E. L. Bavin , et al. 2017. “Language Outcomes at 7 Years: Early Predictors and Co‐Occurring Difficulties.” Pediatrics 139, no. 3: e20161684. 28179482 10.1542/peds.2016-1684

[jlcd70304-bib-0204] McLeod, S. , L. J. Harrison , and C. Wang . 2019. “A Longitudinal Population Study of Literacy and Numeracy Outcomes for Children Identified With Speech, Language, and Communication Needs in Early Childhood.” Early Childhood Research Quarterly 47: 507–517. 10.1016/j.ecresq.2018.07.004.

[jlcd70304-bib-0073] Melvin, S. A. , J. Wray , F. Roberts , J. Pryor , M. Souto‐Manning , and E. Yeung . 2021. “The Home Learning Environment Questionnaire (HLEQ): A Measure of the Quality and Quantity of Activities and Resources in the Home to Support School Readiness.” Educational Review 73, no. 6: 745–772. 10.1080/00131911.2019.1642307.

[jlcd70304-bib-0075] Mettler, H. M. , S. L. Neiling , C. R. Figueroa , N. Evans‐Reitz , and M. Alt . 2023. “Vocabulary Acquisition and Usage for Late Talkers: The Feasibility of a Caregiver‐Implemented Telehealth Model.” Journal of Speech, Language, and Hearing Research 66, no. 1: 257–275. 10.1044/2022_JSLHR-22-00285. PMC10023173 36580564

[jlcd70304-bib-0205] Moher, D. , A. Liberati , J. Tetzlaff , and D. G. Altman . 2009. “Preferred Reporting Items for Systematic Reviews and Meta‐Analyses: The PRISMA Statement.” *BMJ*. 339. PMC3090117 21603045

[jlcd70304-bib-0076] Mol, S. E. , A. G. Bus , M. T. De Jong , and D. J. Smeets . 2008. “Added Value of Dialogic Parent‐Child Book Readings: A Meta‐Analysis.” Early Education and Development 19, no. 1: 7–26.

[jlcd70304-bib-0077] Moore, H. W. , E. E. Barton ,, and M. Chironis . 2014. “A Program for Improving Toddler Communication through Parent Coaching.” Topics in Early Childhood Special Education 33, no. 4: 212–224.

[jlcd70304-bib-0078] Morgan, P. L. , G. Farkas , M. M. Hillemeier , H. Li , W. H. Pun , and M. Cook . 2017. “Cross‐Cohort Evidence of Disparities in Service Receipt for Speech or Language Impairments.” Exceptional Children 84, no. 1: 27–41. 10.1177/0014402917718341.

[jlcd70304-bib-0079] Neiling, S. L. , and C. A. Cutshaw . 2023. “In Support of a Public Health Approach to Late Talking.” American Journal of Speech‐Language Pathology 32, no. 3: 1376–1382. 37105916 10.1044/2023_AJSLP-22-00357

[jlcd70304-bib-0080] Nielsen, D. , K. d'Apice , R. W. Cheung , et al. 2023. “A Randomised Controlled Feasibility Trial of an Early Years Language Development Intervention: Results of the ‘Outcomes of Talking Together Evaluation and Results’ (oTTer) Project.” Pilot and Feasibility Studies 9, no. 1: 107. 10.1186/s40814-023-01333-y. 37386614 PMC10308722

[jlcd70304-bib-0081] Nilsen, P. , J. W. Kirk , K. U. Gunnarsson , and K. Thomas . 2025. “Tempering Implementation Optimism: Distinguishing Between Efficacy and Effectiveness in Implementation Research.” Implementation Science Communications 6, no. 1: 90. 40849653 10.1186/s43058-025-00781-2 PMC12374263

[jlcd70304-bib-0082] Norbury, C. F. , D. Gooch , C. Wray , et al. 2016. “The Impact of Nonverbal Ability on Prevalence and Clinical Presentation of Language Disorder: Evidence From a Population Study.” Journal of Child Psychology and Psychiatry 57, no. 11: 1247–1257. 27184709 10.1111/jcpp.12573 PMC5082564

[jlcd70304-bib-0083] Norbury, C. F. , S. Griffiths , G. Vamvakas , et al. 2021. *Socioeconomic Disadvantage Is Associated With Prevalence of Developmental Language Disorders, but not Rate of Language or Literacy Growth in Children From 4 to 11 Years: Evidence From the Surrey Communication and Language in Education Study (SCALES)*. SSRN. 10.2139/ssrn.3814832.

[jlcd70304-bib-0084] Olson, K. B. , C. L. Wilkinson , M. J. Wilkinson , J. Harris , and A. Whittle . 2016. “Texts for Talking: Evaluation of a Mobile Health Program Addressing Speech and Language Delay.” Clinical Pediatrics 55, no. 11: 1044–1049. 10.1177/0009922816664721. 27554765

[jlcd70304-bib-0085] O'Neill, D. 2009. Language Use Inventory. Knowledge in Development.

[jlcd70304-bib-0086] O'Neill, H. , C.‐A. Murphy , and S. Chiat . 2019. “What Our Hands Tell Us: A Two‐Year Follow‐Up Investigating Outcomes in Subgroups of Children With Language Delay.” Journal of Speech, Language, and Hearing Research 62, no. 2: 356–366. 10.1044/2018_JSLHR-L-17-0261 30950692

[jlcd70304-bib-0087] O'Toole, C. , R. Lyons , and C. Houghton . 2021. “A Qualitative Evidence Synthesis of Parental Experiences and Perceptions of Parent‐Child Interaction Therapy for Preschool Children With Communication Difficulties.” Journal of Speech, Language, and Hearing Research 64, no. 8: 3159–3185. 10.1044/2021_JSLHR-20-00732 34289311

[jlcd70304-bib-0088] Pace, A. , R. Luo , K. Hirsh‐Pasek , and R. M. Golinkoff . 2017. “Identifying Pathways Between Socioeconomic Status and Language Development.” Annual Review of Linguistics 3: 285–308.

[jlcd70304-bib-0089] Page, M. J. , J. E. McKenzie , P. M. Bossuyt , et al. 2021. “The PRISMA 2020 Statement: An Updated Guideline for Reporting Systematic Reviews.” BMJ 372: n71. 33782057 10.1136/bmj.n71 PMC8005924

[jlcd70304-bib-0090] Peredo, T. N. , J. Mancilla‐Martinez , K. Durkin , and A. P. Kaiser . 2022. “Teaching Spanish‐Speaking Caregivers to Implement EMT en Espanol: A Small Randomized Trial.” Early Childhood Research Quarterly 58: 208–219. 10.1016/j.ecresq.2021.08.004. 35058673 PMC8765734

[jlcd70304-bib-0091] Polanin, J. R. , and B. Snilstveit . 2016. “Converting Between Effect Sizes.” Campbell Systematic Reviews 12, no. 1: 1–13.

[jlcd70304-bib-0092] Putnick, D. L. , M. H. Bornstein , S. Eryigit‐Madzwamuse , and D. Wolke . 2017. “Long‐Term Stability of Language Performance in Very Preterm, Moderate‐Late Preterm, and Term Children.” Journal of Pediatrics 181: 74–79.e3. 10.1016/j.jpeds.2016.09.006. 27745750 PMC5274586

[jlcd70304-bib-0093] Quinn, E. D. , A. P. Kaiser , and J. Ledford . 2021. “Hybrid Telepractice Delivery of Enhanced Milieu Teaching: Effects on Caregiver Implementation and Child Communication.” Journal of Speech Language and Hearing Research 64, no. 8: 3074–3099. 10.1044/2021_JSLHR-20-00430. PMC9128738 34289320

[jlcd70304-bib-0094] Rajesh, V. , and L. Venkatesh . 2019. “Preliminary Evaluation of a Low‐Intensity Parent Training Program on Speech‐Language Stimulation for Children With Language Delay.” International Journal of Pediatric Otorhinolaryngology 122: 99–104. 10.1016/j.ijporl.2019.03.034. 30991208

[jlcd70304-bib-0095] Rakhlin, N. , N. Li , D. Momotenko , et al. 2024. “Social Disadvantage and Language Disorder: Is Dissociating Possible?” American Journal of Speech-Language Pathology , 1–18. 10.2139/ssrn.4985214. 42334980

[jlcd70304-bib-0096] Rautakoski, P. , P. af Ursin , A. S. Carter , A. Kaljonen , A. Nylund , and P. Pihlaja . 2021. “Communication Skills Predict Social‐Emotional Competencies.” Journal of Communication Disorders 93: 106138. 34182379 10.1016/j.jcomdis.2021.106138

[jlcd70304-bib-0097] RCSLT . 2019. Developmental Language Disorder Research Priority Setting Partnership Report . Royal College of Speech and Language Therapists. https://www.rcslt.org/wp‐content/uploads/media/Project/RCSLT/DLD‐Report.pdf.

[jlcd70304-bib-0098] RCSLT . 2020. DLD—Questions on Diagnosis and Support . Royal College of Speech and Language Therapists. https://www.rcslt.org/speech‐and‐language‐therapy/clinical‐information/developmental‐language‐disorder/dld‐diagnosis‐and‐support/.

[jlcd70304-bib-0099] Reed, J. L. , S. A. Prince , C. A. Cole , et al. 2014. “Workplace Physical Activity Interventions and Moderate‐to‐Vigorous Intensity Physical Activity Levels Among Working‐Age Women: A Systematic Review Protocol.” Systematic Reviews 3: 147. 25526769 10.1186/2046-4053-3-147 PMC4290810

[jlcd70304-bib-0100] Reilly, S. , and C. McKean . 2023. “Creating the Conditions for Robust Early Language Development for All—Part 1: Evidence‐Informed Child Language Surveillance in the Early Years.” International Journal of Language and Communication Disorders 58: 2222–2241. 10.1111/1460-6984.12929. 37432035

[jlcd70304-bib-0101] Renfrew, C. 2003. Action Picture Test. Speechmark Publishing.

[jlcd70304-bib-0102] Roberts, M. Y. 2014. Using Emprical Benchmarks to Assess the Effects of a Parentimplemented Language Intervention for Children With Language Impairments. ProQuest Information & Learning.

[jlcd70304-bib-0103] Roberts, M. Y. , and A. P. Kaiser . 2011. “The Effectiveness of Parent‐Implemented Language Interventions: A Meta‐Analysis.” American Journal of Speech‐Language Pathology 20, no. 3: 180–199. 10.1044/1058-0360(2011/10-0055). 21478280

[jlcd70304-bib-0104] Roberts, M. Y. , A. P. Kaiser , C. E. Wolfe , J. D. Bryant , and A. M. Spidalieri . 2014. “Effects of the Teach‐Model‐Coach‐Review Instructional Approach on Caregiver Use of Language Support Strategies and Children's Expressive Language Skills.” Journal of Speech, Language, and Hearing Research 57, no. 5: 1851–1869. 10.1044/2014_JSLHR-L-13-0113 24950492

[jlcd70304-bib-0105] Roberts, M. Y. , and A. P. Kaiser . 2015. “Early Intervention for Toddlers With Language Delays: A Randomized Controlled Trial.” Pediatrics 135, no. 4: 686–693. 25733749 10.1542/peds.2014-2134 PMC4379460

[jlcd70304-bib-0106] Roberts, M. Y. , P. R. Curtis , B. J. Sone , and L. H. Hampton . 2019. “Association of Parent Training With Child Language Development: A Systematic Review and Meta‐Analysis.” JAMA Pediatrics 173, no. 7: 671–680. 10.1001/jamapediatrics.2019.1197. 31107508 PMC6537769

[jlcd70304-bib-0107] Romski, M. , L. Adamson , M. Cheslock , and R. Sevcik . 2000. Parent Perception of Language Development (PPOLD). Georgia State University.

[jlcd70304-bib-0206] Roy, P. , H. Kersley , and J. Law . 2005. “The Sure Start Language Measure Standardisation Study.” http://tna.europarchive.org/20070101101348/.

[jlcd70304-bib-0108] Sante, M. , and L. Potvin . 2022. “We Need to Talk About Social Inequalities in Language Development.” American Journal of Speech‐Language Pathology 31, no. 4: 1894–1897. 10.1044/2022_AJSLP-21-00326. 35442715

[jlcd70304-bib-0109] Sawyer, B. , A. Hindman , J. Smith , C. S. Hammer , and J. Santoro . 2025. “Parents Plus: A Parent‐Implemented Intervention for Preschool Children With Developmental Language Disorders.” Language, Speech, and Hearing Services in Schools 56, no. 1: 177–193. 10.1044/2024_LSHSS-24-00042. 39589287

[jlcd70304-bib-0110] Schwab, J. F. , and C. Lew‐Williams . 2016. “Language Learning, Socioeconomic Status, and Child‐Directed Speech.” Wiley Interdisciplinary Reviews: Cognitive Science 7, no. 4: 264–275. 27196418 10.1002/wcs.1393 PMC5901657

[jlcd70304-bib-0111] Semel, E. , E. Wiig , and W. Secord . 2006. Child Evaluation of Language Fundamentals‐Preschool UK. Pearson Assessment.

[jlcd70304-bib-0112] Senent‐Capuz, N. , I. Baixauli‐Fortea , and C. Moret‐Tatay . 2021. “Parent‐Implemented Hanen Program It Takes Two to Talk(R): An Exploratory Study in Spain.” International Journal of Environmental Research and Public Health 18, no. 15: 8214. 10.3390/ijerph18158214. 34360506 PMC8345947

[jlcd70304-bib-0300] Sentenac, M. , S. Johnson , M.‐L. Charkaluk , et al. 2020. “Maternal Education and Language Development at 2 years Corrected age in Children born very Preterm: Results from a European Population‐based Cohort Study.” Journal of Epidemiology and Community Health 74, no. 4: 346–353. 31996408 10.1136/jech-2019-213564

[jlcd70304-bib-0113] Singer, J. 1998. Odd People in: The Birth of Community amongst People on the Autistic Spectrum: A Personal Exploration of a New Social Movement Based on Neurological Diversity. Faculty of Humanities and Social Science University of Technology.

[jlcd70304-bib-0114] Snow, P. , S. Leitão , and N. Kippin . 2021. “Language and Literacy in the Context of Early Life adversity.” In The Handbook of Language and Speech Disorders, edited by J. S. Damico N. Müller , and M. J. Ball 2nd ed., 266–285. Wiley‐Blackwell.

[jlcd70304-bib-0115] Snowling, M. J. , S. E. Stothard , P. Clarke , et al. 2009. YARC York Assessment of Reading for Comprehension Passage Reading. GL Publishers.

[jlcd70304-bib-0116] Snowling, M. J. , S. E. Stothard , and P. J. Clarke . 2012. York Assessment of Reading for Comprehension: Complete Set. GL Assessment.

[jlcd70304-bib-0117] Subba Rao, T. 1992. Manual on Developing Communication Skills in Mentally Retarded Persons. National Institute for the Mentally Handicapped.

[jlcd70304-bib-0118] Suttora, C. , M. Zuccarini , A. Aceti , L. Corvaglia , A. Guarini , and A. Sansavini . 2021. “The Effects of a Parent‐Implemented Language Intervention on Late‐Talkers' Expressive Skills: The Mediational Role of Parental Speech Contingency and Dialogic Reading Abilities.” Frontiers in Psychology 12: 723366. 10.3389/fpsyg.2021.723366. 34566804 PMC8459088

[jlcd70304-bib-0119] Tang, L. , J. Z. Zhao , T. Y. He , et al. 2024. “Effect of Online Parent Training in Promoting Language Development of Children With Language Delay in Hubei Province, China.” International Journal of Language & Communication Disorders 59: 1322–1335. 10.1111/1460-6984.12945. 38165073

[jlcd70304-bib-0120] Thomas‐Meyer, M. , C. Harding , and J. Wilson . 2018. “Focus on the Outcomes of Communication under Six (FOCUS‐34): A Valid and Reliable Tool for Evaluating Pediatric Speech‐Language Outcomes.” Journal of Communication Disorders 76: 69–83.

[jlcd70304-bib-0121] Thomas‐Meyer, M. , R. Bornstein , and I. L. Sucharski . 2019. “Creating the ‘Maternal Object Relations Scale’ (MORS): A New Tool to Assess Mother‐Child Relationships.” Infant Mental Health Journal 40, no. 2: 219–234.

[jlcd70304-bib-0122] Thomas‐Stonell, N. , B. Robertson , J. Walker , B. Oddson , K. Washington , and P. Rosenbaum . 2012. FOCUS∖Copyright: Focus on the Outcomes of Communication Under Six. Holland Bloorview Kids Rehabilitation Hospital.

[jlcd70304-bib-0123] Thordardottir, E. , and S. Topbaş . 2021. “How Aware Is the Public of the Existence, Characteristics and Causes of Language Impairment in Childhood and Where Have They Heard About It? A European Survey.” Journal of Communication Disorders 89: 106057. 33279754 10.1016/j.jcomdis.2020.106057

[jlcd70304-bib-0124] Thorlund, K. , G. Imberger , B. C. Johnston , et al. 2012. “Evolution of Heterogeneity (I2) Estimates and Their 95% Confidence Intervals in Large Meta‐Analyses.” PLoS ONE 7, no. 7: e39471. 22848355 10.1371/journal.pone.0039471 PMC3405079

[jlcd70304-bib-0125] Tomblin, J. B. , N. L. Records , P. Buckwalter , X. Zhang , E. Smith , and M. O'Brien . 1997. “Prevalence of Specific Language Impairment in Kindergarten Children.” Journal of Speech, Language, and Hearing Research 40, no. 6: 1245–1260. 10.1044/jslhr.4006.1245 PMC5075245 9430746

[jlcd70304-bib-0126] Sandwell Primary Care Trust . 2012. Wellcomm: A Speech and Language Toolkit for the Early Years. Granada Learning/NFER‐Nelson.

[jlcd70304-bib-0127] U.P.I.A.S . 1976. Fundamental Principles of Disability. Union of the Physically Impaired Against Segregation.

[jlcd70304-bib-0128] Valavani, E. , M. Blesa , P. Galdi , et al. 2022. “Language Function Following Preterm Birth: Prediction Using Machine Learning.” Pediatric Research 92, no. 2: 480–489. 10.1038/s41390-021-01779-x. 34635792 PMC8503721

[jlcd70304-bib-0129] Vally, Z. , L. Murray , M. Tomlinson , and P. J. Cooper . 2015. “The Impact of Dialogic Book‐Sharing Training on Infant Language and Attention: A Randomized Controlled Trial in a Deprived South African Community.” Journal of Child Psychology and Psychiatry 56, no. 8: 865–873. 10.1111/jcpp.12352. 25399699 PMC4552334

[jlcd70304-bib-0130] Vrinda, R. , and S. K. Kunnath . 2023. “A Pilot Study to Evaluate the Effectiveness of Parent Mediated Group Intervention Program for Developmental Language Disorders.” Clinical Archives of Communication Disorders 8, no. 2: 47–56.

[jlcd70304-bib-0131] Wake, M. , P. Levickis , S. Tobin , et al. 2015. “Two‐Year Outcomes of a Population‐Based Intervention for Preschool Language Delay: An RCT.” Pediatrics 136, no. 4: e838–e847. 10.1542/peds.2015-1337. 26347428

[jlcd70304-bib-0133] Wallace, B. C. , I. J. Dahabreh , T. A. Trikalinos , J. Lau , P. Trow , and C. H. Schmid . 2012. “Closing the Gap Between Methodologists and End‐Users: R as a Computational Back‐End.” Journal of Statistical Software 49: 1–15.

[jlcd70304-bib-0134] World Health Organization . 2021. Social Determinants of Health. WHO. https://www.who.int/teams/social‐determinants‐of‐health.

[jlcd70304-bib-0135] World Health Organization . 2022. ICD‐11: International Classification of Diseases. WHO. https://icd.who.int/.

[jlcd70304-bib-0400] Zerbeto, A. B. , F. M. Cortelo , and É. B. C. Filho . 2015. “Association Between Gestational Age and Birth Weight on the Language Development of Brazilian Children: A Systematic Review.” Jornal de Pediatria 91, no. 4: 326–332. 25913048 10.1016/j.jped.2014.11.003

[jlcd70304-bib-0136] Zhang, X. , and J. Li . 1994. “The Revise of Gesell Developmental Scale on 31/2–6 Years of Age in Beijing.” Zhongguo Dang Dai Er Ke Za Zhi 2: 148–150.

[jlcd70304-bib-0137] Ziegenfusz, S. , J. Paynter , B. Flückiger , and M. F. Westerveld . 2022. “A Systematic Review of the Academic Achievement of Primary and Secondary School‐Aged Students With Developmental Language Disorder.” Autism & Developmental Language Impairments 7: 23969415221099397. 10.1177/23969415221099397. 36382072 PMC9620692

[jlcd70304-bib-0138] Zimmerman, I. , V. Steiner , and R. Pond . 2002. Preschool Language Scale, 4th ed. Harcourt Assessment.

[jlcd70304-bib-0139] Zuccarini, M. , C. Suttora , A. Bello , et al. 2020. “A Parent‐Implemented Language Intervention for Late Talkers: An Exploratory Study on Low‐Risk Preterm and Full‐Term Children.” International Journal of Environmental Research and Public Health 17, no. 23: 9123. 10.3390/ijerph17239123. 33297374 PMC7730473

