# Supplementary material for: Effectiveness of Caregiver‐Mediated Spoken Language Interventions for Children Under Five at Risk of Developmental Language Disorder: A Systematic Review and Meta‐Analysis
Source: Int J Lang Commun Disord. 2026 Aug 21;61(5):e70304. doi: 10.1111/1460-6984.70304 (PMC13496101; doi:10.1111/1460-6984.70304)
Supplement: Supplementary file 1 [file JLCD-61-0-s001.docx]

|  | Inclusion criteria | Exclusion criteria |
| --- | --- | --- |
| Population | Children under 5 years of age  Presenting with risk factors for Developmental Language Disorder including delayed expressive language, family socioeconomic stressors, prematurity, behavioural difficulties, or family history of speech, language and communication needs  Any region | Studies specifically addressing bilingualism  Children with chromosomal, metabolic, progressive or acquired cognitive/neurological disorders  Children with deafness or hearing loss  Children with an existing diagnosis of intellectual disability  Children with a diagnosis of autism or where the stated primary difficulty is social communication |
| Intervention | Caregiver- or primary caregiver-mediated oral language intervention delivered indirectly via healthcare professionals (e.g. Speech and Language Therapists, Speech and Language Therapy Assistants) involving face-to-face or online, individual or group caregiver coaching in techniques to promote language development in everyday life | Intervention delivered directly by healthcare or education professionals  Behavioural or wellbeing interventions  Universal/targeted written advice or signposting only, without an individualised coaching and modelling component |
| Comparator/ Control | No intervention, intervention unrelated to language development, 'wait and see' approaches, universal/targeted advice only, or 'treatment as usual' without a stated caregiver-mediated component | Healthcare or education professional delivered direct intervention  'Treatment as usual' with a defined caregiver-mediated component |
| Outcome | Language assessment gains  Child participation, wellbeing or quality of life  Educational attainment and cognitive development  Caregiver self-efficacy or adherence to strategies | Economic effects, efficiency, or cost of service delivery  Caregiver quality of life or caregiver stress  Caregiver engagement or retention in intervention |
