# Supplementary material for: Effectiveness of Caregiver‐Mediated Spoken Language Interventions for Children Under Five at Risk of Developmental Language Disorder: A Systematic Review and Meta‐Analysis
Source: Int J Lang Commun Disord. 2026 Aug 21;61(5):e70304. doi: 10.1111/1460-6984.70304 (PMC13496101; doi:10.1111/1460-6984.70304)
Supplement: Supplementary file 2 [file JLCD-61-0-s002.docx]

# Appendix: Outcome measures included in meta-analysis

| Study | Language constructs measured | Assessments used |
| --- | --- | --- |
| (Buschmann et al., 2015) | Expressive vocabulary  Plural forming  Sentence comprehension | Expressive Vocabulary Test for 3–5-Year‑Olds ^C E^ (AWST‑R) (Kiese-Himmel, 2005)  SETK 3‑5 Plural Forming ^C E^, Sentence Comprehension ^C R^ (Grimm, 2000) |
| (Roberts & Kaiser, 2015) | Expressive communication  Auditory comprehension  Expressive vocabulary | Preschool Language Scale – Fourth Edition (PLS‑4) Expressive Communication ^C E^, Auditory Comprehension subscales ^C R^  (Zimmerman et al., 2002);  Peabody Picture Vocabulary Test – Fourth Edition ^C R^ (PPVT‑4) (Dunn & Dunn, 1981);  MacArthur–Bates Communicative Development Inventories (CDI): Words & Sentences ^C R E^ (Fenson et al., 2006)  Expressive One-Word Picture Vocabulary Test ^C R E^ – Fourth Edition (EOWPVT‑4) (Brownell, 2000) |
| (Vally et al., 2015) | Receptive vocabulary  Expressive vocabulary | MacArthur–Bates Communicative Development Inventories ^C R^ (CDI) (translated into isiXhosa by study team) (Fenson et al., 2000);  Adapted receptive language measure ^C R^ modelled on Peabody Picture Vocabulary Test – Revised (PPVT‑R) (Dunn & Dunn, 1981) |
| (Wake et al., 2015) | Expressive communication  Auditory comprehension  Expressive vocabulary  Sentence use  Language use/complexity | Preschool Language Scale - Fourth Edition (PLS‑4): auditory comprehension ^C R^ and expressive communication ^C E^ (Zimmerman et al., 2002)  MacArthur–Bates Communicative Development Inventories ^C^ (MCDI-III) (Fenson et al., 2006)  Sure Start language measure ^C E^ (Roy et al., 2005)  Expressive Vocabulary Test ^C E^ (EVT) (not cited by authors) |
| (Bagner et al., 2016) | Number of different utterances spoken (‘types’; lexical diversity of utterances/words produced during a 5-minute infant-led play observation)  Number of total utterances spoken (‘tokens’; total count of word approximations and words produced during the observation) | Observational language sample analysed with Child Language Data Exchange System ^C^  (CHILDES) (MacWhinney, 2000) |
| (Burgoyne et al., 2018) | Expressive vocabulary  Receptive vocabulary  Expressive grammar  Receptive grammar  Listening comprehension | CELF Preschool II UK – Expressive Vocabulary ^C E^ and Sentence Structure ^C E^ subtests (Semel et al., 2006)  Renfrew Action Picture Test (APT) Information ^C E^ and Grammar ^C E^ subscales (Renfrew, 2003)  BPVS3 – British Picture Vocabulary Scale ^C R^, 3rd Ed.(Dunn et al., 2009)  York Assessment of Reading for Comprehension (YARC) stories ^C R^  (C. Hulme et al., 2009) (adapted)  Informal observational measure of story retelling: total words ^C E^, number of different words ^C E^ and MLU ^C E^ measures. |
| (Nielsen et al., 2023) | Expressive language  Receptive language | Oxford Communication Development Inventory – Short (CDI‑Short); Understanding ^C R^ and Speaking ^C E^ measures (Hamilton et al., 2000)  WellComm Language Assessment ^C E^ (Sandwell Primary Care Trust, 2012) |
| (McGowan et al., 2024) | Receptive language  Expressive language | Bayley Scales of Infant and Toddler Development, Third Edition (BSID‑III): Receptive ^C R^  and Expressive ^C E^ Language Subscales (Bayley, 2006) |
| (Sawyer et al., 2025) | Overall language ability  Expressive grammar  Expressive vocabulary  Expressive morphosyntax  Mean Length of Utterance in morphemes (MLU-m)  Number of different words | CELF Preschool -Second Edition (CELF Preschool–2) Core Language Index ^C^, Sentence Structure ^E^, Word Structure ^E^ and Expressive Vocabulary ^E^ subtests (Wiig et al., 2004);  Test of Early Grammatical Impairment and researcher-created morphosyntax probes, to create morphosyntax composite score ^C E^  Manualised coding of child Mean Length of Utterance ^C E^ and number of different words ^C E^ |

Key: Used in meta-analysis of ^C^  composite, ^R^  receptive and  ^E^ expressive language scores
