# Supplementary material for: Effectiveness of Caregiver‐Mediated Spoken Language Interventions for Children Under Five at Risk of Developmental Language Disorder: A Systematic Review and Meta‐Analysis
Source: Int J Lang Commun Disord. 2026 Aug 21;61(5):e70304. doi: 10.1111/1460-6984.70304 (PMC13496101; doi:10.1111/1460-6984.70304)
Supplement: Supplementary file 3 [file JLCD-61-0-s003.docx]

**Identification of studies via databases**

Records removed *before screening*:

Duplicate records removed (n = 64)

Records identified from*:

Databases (n = 299)

Hand-searching reference lists (n = 3)

**Identification**

Records screened

(n = 235)

Records excluded**

(n = 173)

Reports sought for retrieval

(n = 62)

Reports not retrieved

(n = 0)

**Screening**

Reports excluded (n = 36):

Wrong outcomes= 6

Wrong control= 1

Wrong comparator= 7

Wrong intervention= 7

Wrong study design= 5

Wrong patient population= 10

Reports assessed for eligibility

(n = 62)

Studies included in qualitative synthesis (n = 26)

Studies also included in meta-analysis (n = 9)

**Included**

*Consider, if feasible to do so, reporting the number of records identified from each database or register searched (rather than the total number across all databases/registers).

**If automation tools were used, indicate how many records were excluded by a human and how many were excluded by automation tools.

Source: Page MJ, et al. BMJ 2021;372:n71. doi: 10.1136/bmj.n71.

This work is licensed under CC BY 4.0. To view a copy of this license, visit <https://creativecommons.org/licenses/by/4.0/>
