# Supplementary material for: Effectiveness of Caregiver‐Mediated Spoken Language Interventions for Children Under Five at Risk of Developmental Language Disorder: A Systematic Review and Meta‐Analysis
Source: Int J Lang Commun Disord. 2026 Aug 21;61(5):e70304. doi: 10.1111/1460-6984.70304 (PMC13496101; doi:10.1111/1460-6984.70304)
Supplement: Supplementary file 4 [file JLCD-61-0-s004.docx]

## Appendix: Study characteristics

| Study  Country | Study design | Population (N)  (Intervention/  Control) | DLD risk factor | Intervention  (Control) | Mode of delivery  Delivered by | Outcomes | Outcome measures | Quality/27 |
| --- | --- | --- | --- | --- | --- | --- | --- | --- |
| (Akemoglu et al., 2022)  USA | Single-case multiple baseline design | **3**  (n/a) | Language delay and/or developmental delay in Individualized Family Service Plan (IFSP) | **Parent-Implemented Communication Strategies– Storybook (i-PiCSS)**  (n/a) | **Online**  Research assistant | Language assessment gains (observational/ informal assessment); Caregiver adherence to strategies | Informal observational measures of child communication behaviours; Observational coding of caregiver fidelity to and rate of strategy use | Fair (15) |
| (Bagner et al., 2016)  USA | RCT | **60**  (31/29) | Behavioural difficulties | **Infant Behaviour Programme**  (Standard paediatric care) | **Face to face**  Research assistant | Informal language assessment gains | Child Language Data Exchange System (CHILDES) (MacWhinney, 2000): Total utterances (tokens),  Different utterances (types) | Good (20) |
| (Burgoyne et al., 2018)  United Kingdom | RCT | **208**  (103/105) | Socioeconomic factors (targeted areas) | **Parent-mediated early language enrichment programme**  (Movement skills and self-care programme) | **Face to face**  Trained children’s centre staff | Formal and informal language assessment gains | CELF Preschool II UK – Expressive Vocabulary and Sentence Structure subtests (Semel et al., 2006); Renfrew Action Picture Test (APT)(Renfrew, 2003); BPVS3 – British Picture Vocabulary Scale, 3rd Ed.(L. Dunn et al., 2009); York Assessment of Reading for Comprehension (YARC) stories (Snowling et al., 2009) (adapted) informal observational measure of story retelling; York Assessment of Reading Comprehension (YARC) - Early Word Recognition (Snowling et al., 2012) | Excellent (24) |
| (Buschmann et al., 2015)  Germany | RCT | **43**  (23/20) | Language concern reported by caregiver / Screening tool (MacArthur Bates) | **Heidelberg Parent-Based Language Intervention (HPLI)**  (Treatment as usual with direct SLT) | **Face to face**  (not recorded) | Formal language assessment | Expressive Vocabulary Test for 3–5-Year‑Olds (AWST‑R) (Kiese-Himmel, 2005); SETK 3‑5 Plural Forming, Sentence Comprehension, Word Span, Non-word Repetition, Sentence Repetition subtests (Grimm, 2001); K‑ABC Number Recall, Word Order subtests (Kaufman & Kaufman, 1983) | Excellent (26) |
| (Cunningham et al., 2019)  Canada | Case series | **24**  (n/a) | Language concern reported by caregiver | **Target Word™, The Hanen Program® for Parents of Children who are Late Talkers** | **Face to face**  Speech and Language Therapist | Formal and  Informal language assessment gains,  Consonant inventory | FOCUS‑34 (Focus on the Outcomes of Communication Under Six) (Thomas-Stonell et al., 2012);  Communication Function Classification System (CFCS) (Hidecker et al., 2017); MacArthur–Bates Communicative Development Inventories – Words & Gestures (MBCDI) (Fenson et al., 2006)  Informal checklist assessing consonant inventory | Fair (16) |
| (Cycyk et al., 2020)  USA | Case series | **8**  (n/a) | Language concern reported by caregiver | **Language and Play Every Day (LAPE)** | **Face to face**  Student research assistants supervised by SLTs | Informal language assessment gains  Parent self-efficacy/confidence in supporting language development | MacArthur–Bates Communicative Development Inventories (MCDI): Words and Gestures (Spanish and English versions) (Fenson et al., 2006; Jackson-Maldonado et al., 2021);  Systematic coding of caregiver use of communication‑ facilitating strategies, child rate of communication, child rate of initiation; Caregiver survey | Fair (16) |
| (Falkus et al., 2016)  United Kingdom | Case series | **18**  (n/a) | Language concern reported by caregiver | **Parent–child interaction therapy (PCIT)** | **Face to face**  Speech and Language Therapist | Formal and  Informal language assessment gains | Child Mean Length of Utterance; Observational scale of caregiver interaction strategies (e.g., commenting, waiting, praising); Ratio of child to parent speech | Fair (18) |
| (McDonald et al., 2019)  United Kingdom | Case series | **7**  (n/a) | Language concern reported by caregiver | **Home Talk intervention** | **Face to face**  Speech and Language Therapy assistant | Formal language assessment gains | Language Use Inventory (LUI) – Words subscale (O’Neill, 2009) | Poor (13) |
| (McGowan et al., 2024)  USA | RCT | **64**  (32/33) | Prematurity | **Caregiver-mediated early language enrichment programme**  (Infant safety programme) | **Face to face**  Research assistant | Formal language assessment gains, Cognitive skill gains | Bayley Scales of Infant and Toddler Development, Third Edition (BSID‑III) (Bayley, 2006) | Excellent (25) |
| (Mettler et al., 2023)  USA | Feasibility study | **5**  (n/a) | Language concern reported by caregiver | **Caregiver-implemented telehealth model of Vocabulary Acquisition and Usage for Late Talkers (VAULT)** | **Online**  Speech and Language Therapist | Formal language assessment, Caregiver confidence, engagement with programme, adherence to strategies | MacArthur–Bates Communicative Development Inventories (CDI): Words & Sentences (Spanish and English versions) (Fenson et al., 2006; Jackson-Maldonado et al., 2021); Caregiver interviews and surveys | Fair (17) |
| (Moore et al., 2014)  USA | Case series | **8**  (n/a) | Standardised language score | **Language and Play Everyday (LAPE)** | **Face to face**  Speech and Language Therapist | Formal and informal language assessment | Preschool Language Scale—Fourth Edition (PLS‑4) (Zimmerman et al., 2002); MacArthur–Bates Communicative Development Inventory (CDI‑II) (Fenson et al., 2006); Child Mean Length of Utterance; Frequency of child communication behaviours from coded video samples | Fair (16) |
| (Nielsen et al., 2023)  United Kingdom | RCT | **102**  (52/50) | Socioeconomic factors (eg targeted areas) | **Talking Together**  (Waitlist control, usual care and input reported) | **Face to face**  Language Development Workers | Formal language assessment gains | Oxford Communication Development Inventory – Short (CDI‑Short) (Hamilton et al., 2000); WellComm Language Assessment (Sandwell Primary Care Trust, 2012) | Fair (18) |
| (Olson et al., 2016)  USA | Case series | **31**  (n/a) | Socioeconomic factors (eg targeted areas) | **uTALK program: Text messaging programme promoting parent mediated language development strategies**  (n/a) | **Text message**  Fully automated via text | Parent self-efficacy/confidence in supporting language development | Parent surveys and interviews | Fair (18) |
| (Peredo et al., 2022)  USA | Intent-to-Treat RCT | **20**  (10/10) | Language concern reported, Standardised language score | **EMT en Español via Teach-Model-Coach-Review approach**  (Waitlist control) | **Face to face**  One SLT, one researching Psychotherapist | Formal language assessment;  Caregiver adherence to strategies | Manualised coding of caregiver use of strategies during interactions | Good (23) |
| (Quinn et al., 2021)  USA | Case series | **4**  (n/a) | Standardised language score | **Enhanced Milieu Teaching (EMT)**  (n/a) | **40% in-person and 60% telepractice sessions**  Speech and Language Therapist | Informal language assessment gains;  Caregiver adherence to strategies | Count measures of child communication acts, Number of different words;  Coding of caregiver implementation fidelity and frequency of strategy use | Fair (16) |
| (Rajesh & Venkatesh, 2019)  India | Case series | **9**  (n/a) | Language concern reported, Standardised language score | **Low intensity caregiver-mediated language program**  (n/a) | **Face to face**  Not reported | Informal language assessment gains; Caregiver adherence to strategies;  Caregiver confidence in supporting language development | Language Assessment Tool (LAT) derived from Subba Rao (1992);  Caregiver‑reported child  language behaviour change; Coding of caregiver-child interaction videos; Caregiver questionnaires | Fair (15) |
| (Roberts et al., 2014)  USA | Case series | **4**  (n/a) | Standardised language score | **Teach-Model-Coach-Review instructional approach**  (n/a) | **Face to face**  Research assistant | Formal and informal language assessment gains;  Caregiver adherence to strategies | Preschool Language Scale – Fourth Edition (PLS‑4) (Zimmerman et al., 2002); Expressive One-Word Picture Vocabulary Test – Fourth Edition (EOWPVT‑4) (Brownell, 2000); Manualised coding of child Mean Length of Utterance, number of different words and total number of words;  Coding of caregiver behaviour from video‑recorded sessions | Fair (15) |
| (Roberts & Kaiser, 2015)  USA | RCT | **97**  (45/52) | Standardised language score | **Enhanced Milieu Teaching (EMT)**  (Waitlist control, usual care and input reported) | **Face to face**  Research assistant | Formal and  Informal language assessment gains; Caregiver adherence to strategies | Preschool Language Scale – Fourth Edition (PLS‑4) (Zimmerman et al., 2002);  Peabody Picture Vocabulary Test – Fourth Edition (PPVT‑4) (L. M. Dunn & Dunn, 2007); MacArthur–Bates Communicative Development Inventories (CDI): Words & Sentences (Fenson et al., 2006); Expressive One-Word Picture Vocabulary Test – Fourth Edition (EOWPVT‑4) (Brownell, 2000);  Observational coding of frequency of caregiver use of strategies | Excellent (24) |
| (Sawyer et al., 2025)  USA | RCT | **31**  (16/15) | **Individualised Educational Plan and working diagnosis of DLD** | **Parents Plus**  (Waitlist control, usual care and input reported) | **Online**  Research assistant | Formal and  informal language assessment gains | CELF Preschool–Second Edition (CELF Preschool–2) Core Language Index (Semel et al., 2006);  Manualised coding of child Mean Length of Utterance, number of different words and total number of words; Researcher-created probe for morphosyntactic skills | Good (19) |
| (Senent-Capuz et al., 2021)  Spain | Quasi-experimental study | **17**  (n/a) | Language concern reported, Standardised language score | **It Takes Two to Talk® - The Hanen Program® for Parents (ITTT)**  (Clinician-directed therapy) | **Face to face**  Speech and Language Therapist | Formal and  informal language assessment gains | MacArthur–Bates Communicative Development Inventories (MCDI-S) (Spanish version) (Martinez, 2007); Reynell Developmental Language Scales (RDLS-III) (Edwards et al., 1999);  Parent Perception of Language Development (PPOLD) (Romski et al., 2000) | Fair (16) |
| (Suttora et al., 2021)  Italy | Quasi-experimental study | **46**  (n/a) | Prematurity | **Caregiver-implemented dialogic reading and focused stimulation techniques**  (Waitlist control) | **Face to face**  Psychologist | Formal language assessment gains | MacArthur–Bates Communicative Development Inventories (CDI): Words & Sentences (Fenson et al., 2006); Manualised coding of child Mean Length of Utterance, word types and word tokens | Good (20) |
| (Tang et al., 2024)  China | Case series | **146**  n/a | Language concern reported, Standardised language score | **Online parent training program: Bethel Hearing and Speaking Training Center Family Training for Early Communication & Language Development (Bethel Family Training Program, BFT)**  (n/a) | **Online**  Speech and Language Therapist | Formal language assessment | Gesell Developmental Schedules (GDS) (Zhang & Li, 1994) | Fair (17) |
| (Vally et al., 2015)  South Africa | RCT | **91**  (49/42) | Socioeconomic factors (eg targeted areas) | **Dialogic book-sharing programme**  (Waitlist control) | **Face to face**  Trained community members | Formal and informal language assessment gains | MacArthur–Bates Communicative Development Inventories (CDI) (translated into isiXhosa by study team) (Fenson et al., 2006);  Adapted receptive language measure modelled on Peabody Picture Vocabulary Test – Revised (PPVT‑R) (L. M. Dunn & Dunn, 2007) | Excellent (24) |
| (Vrinda & Kunnath, 2023)  India | Case series | **8**  (n/a) | Language Disorder provisional diagnosis received | **Caregiver-mediated early language enrichment programme**  (n/a) | **Face to face**  Speech and Language Therapist | Formal language assessment | Assessment of Language Development (ALD) - receptive and expressive language (Lakkanna et al., 2008);  Get Ready to Read (GRTR) (Benmeleh, 2011) | Poor (13) |
| (Wake et al., 2015)  Australia | RCT | **301**  (158/143) | Language concern reported | **Let's Learn Language: modified “You Make the Difference” programme**  (Treatment as usual) | **Face to face**  Three interventionists (one with a speech pathology background and two with psychology backgrounds) | Formal and informal language assessment gains | Preschool Language Scale – Fourth Edition (PLS‑4) – receptive and expressive (Zimmerman et al., 2002); MacArthur–Bates Communicative Development Inventories (MCDI-III) (Fenson et al., 2006)  Sure Start language measure (Roy et al., 2005) | Excellent (25) |
| (Zuccarini et al., 2020)  Italy | Non-randomised controlled trial | **50**  (27/23) | Language concern reported | **Caregiver-implemented dialogic book reading intervention (Girolametto)**  (Waitlist control) | **Face to face**  Psychologist | Informal language assessment gains, Cognitive scores | MacArthur–Bates Communicative Development Inventories (CDI) (Italian version) (Caselli et al., 2015);  Bayley Scales of Infant and Toddler Development, Third Edition (BSID‑III) (Bayley, 2006) | Fair (17) |

References for table

Akemoglu, Y., Hinton, V., Laroue, D., & Jefferson, V. (2022). A Parent-Implemented Shared Reading Intervention via Telepractice. *Journal of Early Intervention 2022 44(2): 190-210*.

Bagner, D. M., Garcia, D., & Hill, R. (2016). Direct and Indirect Effects of Behavioral Parent Training on Infant Language Production. *Behavior Therapy*, *47*(2), 184–197. https://doi.org/10.1016/j.beth.2015.11.001

Bayley, N. (2006). *Bayley Scales of Infant and Toddler Development* (3rd edn). Harcourt Assessment.

Benmeleh, E. (2011). *Get Ready to Read!: Assessing the efficacy of an emergent literacy screening tool*. Nova Southeastern University.

Brownell, R. (2000). *Expressive one-word picture vocabulary test: EOWPVT*. Academic Therapy Publications.

Burgoyne, K., Gardner, R., Whiteley, H., Snowling, M. J., & Hulme, C. (2018). Evaluation of a parent‐delivered early language enrichment programme: Evidence from a randomised controlled trial. *JOURNAL OF CHILD PSYCHOLOGY AND PSYCHIATRY*, *59*(5), 545–555.

Buschmann, A., Multhauf, B., Hasselhorn, M., & Pietz, J. (2015). Long-Term Effects of a Parent-Based Language Intervention on Language Outcomes and Working Memory for Late-Talking Toddlers. *Journal of Early Intervention*, *37*(3), 175–189. https://doi.org/10.1177/1053815115609384

Caselli, M. C., Bello, A., Rinaldi, P., Stefanini, S., & Pasqualetti, P. (2015). Il primo vocabolario del bambino: Gesti, parole e frasi. *Valori Di Riferimento Fra*, *8*, 8–36.

Cunningham, B. J., Kwok, E., Earle, C., & Cardy, J. O. (2019). Exploring participation and impairment-based outcomes for Target Word^TM^: A parent-implemented intervention for preschoolers identified as late-to-talk. *Child Language Teaching & Therapy*, *35*(2), 145–164. https://doi.org/10.1177/0265659019846931

Cycyk, L. M., Moore, H. W., De Anda, S., Huerta, L., Méndez, S., Patton, C., & Bourret, C. (2020). Adaptation of a Caregiver-Implemented Naturalistic Communication Intervention for Spanish-Speaking Families of Mexican Immigrant Descent: A Promising Start. *American Journal of Speech-Language Pathology*, *29*(3), 1260–1282. https://doi.org/10.1044/2020_AJSLP-19-00142

Dunn, L., Dunn, L., Styles, B., & Sewell, J. (2009). British picture vocabulary scale–Third edition (BPVS-III). *National Foundation for Educational Research*.

Dunn, L. M., & Dunn, L. M. (2007). Peabody Picture Vocabulary Test (4th ed.). *PsycTESTS Dataset*.

Edwards, S., Garman, M., Hughes, A., Letts, C., & Sinka, I. (1999). Assessing the comprehension and production of language in young children: An account of the Reynell Developmental Language Scales III. *International Journal of Language & Communication Disorders*, *34*(2), 151–171.

Falkus, G., Tilley, C., Thomas, C., Hockey, H., Kennedy, A., Arnold, T., Thorburn, B., Jones, K., Patel, B., Pimenta, C., Shah, R., Tweedie, F., O’Brien, F., Leahy, R., & Pring, T. (2016). Assessing the effectiveness of parent–child interaction therapy with language delayed children: A clinical investigation. *Child Language Teaching and Therapy 2016 32(1): 7-17*.

Fenson, L., Marchman, V. A., Thal, D. J., Dale, P. S., Reznick, J. S., & Bates, E. (2006). MacArthur-Bates Communicative Development Inventories. *PsycTESTS Dataset*.

Grimm, H. (2001). *Sprachentwicklungstest für drei-bis fünfjährige Kinder: SETK 3-5; Diagnose von Sprachverarbeitungsfähigkeiten und auditiven Gedächtnisleistungen*. Hogrefe, Verlag für Psychologie.

Hamilton, A., Plunkett, K., & Schafer, G. (2000). Infant vocabulary development assessed with a British communicative development inventory. *Journal of Child Language*, *27*(3), 689–705.

Hidecker, M. J. C., Cunningham, B. J., Thomas-Stonell, N., Oddson, B., & Rosenbaum, P. (2017). Validity of the Communication Function Classification System for use with preschool children with communication disorders. *Developmental Medicine & Child Neurology*, *59*(5), 526–530.

Jackson-Maldonado, D., Thal, D., Fenson, L., Marchman, V., Newton, T., & Conboy, B. (2021). *MacArthur inventarios del Desarrollo de habilidades comunicativas (Inventarios): User’s guide and technical manual*.

Kaufman, A. S., & Kaufman, N. L. (1983). Kaufman assessment battery for children. *Psychological Assessment*.

Kiese-Himmel, C. (2005). *AWST-R-Aktiver Wortschatztest für 3-bis 5-jährige Kinder*. Beltz Test Göttingen.

Lakkanna, S., Venkatesh, K., & Bhat, J. (2008). Assessment of language development. *Bakersfield, CA: Omni Therapy Services*.

MacWhinney, B. (2000). *The CHILDES project: Tools for analyzing talk: Volume I: Transcription format and programs, volume II: The database*. MIT Press One Rogers Street, Cambridge, MA 02142-1209, USA journals-info ….

Martinez, M. F. (2007). La evaluación del desarrollo comunicativo y lingüístico mediantela versión española de los inventarios MacArthur-Bates. *Psicothema*.

McDonald, D., Colmer, S., Guest, S., Humber, D., Ward, C., & Young, J. (2019). Parent-implemented language intervention delivered by therapy assistants for two-year-olds at risk of language difficulties: A case series. *Child Language Teaching & Therapy*, *35*(2), 113–124. https://doi.org/10.1177/0265659019842244

McGowan, E. C., Caskey, M., Tucker, R., & Vohr, B. R. (2024). A Randomized Controlled Trial of a Neonatal Intensive Care Unit Language Intervention for Parents of Preterm Infants and 2-Year Language Outcomes. *The Journal of Pediatrics*, *264*, 113740. https://doi.org/10.1016/j.jpeds.2023.113740

Mettler, H. M., Neiling, S. L., Figueroa, C. R., Evans-Reitz, N., & Alt, M. (2023). Vocabulary Acquisition and Usage for Late Talkers: The Feasibility of a Caregiver-Implemented Telehealth Model. *Journal of Speech, Language, and Hearing Research*, *66*(1), 257–275. https://doi.org/10.1044/2022_JSLHR-22-00285

Moore, H. W., Barton, E. E., & Chironis, M. (2014). A Program for Improving Toddler Communication Through Parent Coaching. *Topics in Early Childhood Special Education 2014 33(4): 212-224*.

Nielsen, D., d’Apice, K., Cheung, R. W., Bryant, M., Heald, R., Storr, C., Tracey, L., Rashid, R., Dickerson, J., & Bowyer-Crane, C. (2023). A randomised controlled feasibility trial of an early years language development intervention: Results of the ‘outcomes of Talking Together evaluation and results’ (oTTer) project. *Pilot and Feasibility Studies*, *9*(1), 107. https://doi.org/10.1186/s40814-023-01333-y

Olson, K. B., Wilkinson, C. L., Wilkinson, M. J., Harris, J., & Whittle, A. (2016). Texts for Talking: Evaluation of a Mobile Health Program Addressing Speech and Language Delay. *Clinical Pediatrics*, *55*(11), 1044–1049. https://doi.org/10.1177/0009922816664721

O’Neill, D. (2009). Language use inventory. *Ontario, Canada: Knowledge in Development*, *51*.

Peredo, T. N., Mancilla-Martinez, J., Durkin, K., & Kaiser, A. P. (2022). Teaching spanish-speaking caregivers to implement EMT en Espanol: A small randomized trial. *Early Childhood Research Quarterly*, *58*, 208–219. https://doi.org/10.1016/j.ecresq.2021.08.004

Quinn, E. D., Kaiser, A. P., & Ledford, J. (2021). Hybrid Telepractice Delivery of Enhanced Milieu Teaching: Effects on Caregiver Implementation and Child Communication. *JOURNAL OF SPEECH LANGUAGE AND HEARING RESEARCH*, *64*(8), 3074–3099. https://doi.org/10.1044/2021_JSLHR-20-00430

Rajesh, V., & Venkatesh, L. (2019). Preliminary evaluation of a low-intensity parent training program on speech-language stimulation for children with language delay. *International Journal of Pediatric Otorhinolaryngology*, *122*, 99–104. https://doi.org/10.1016/j.ijporl.2019.03.034

Renfrew, C. (2003). *Action Picture Test*. Speechmark Publishing.

Roberts, M. Y., & Kaiser, A. P. (2015). Early intervention for toddlers with language delays: A randomized controlled trial. *Pediatrics*, *135*(4), 686–693.

Roberts, M. Y., Kaiser, A. P., Wolfe, C. E., Bryant, J. D., & Spidalieri, A. M. (2014). Effects of the teach-model-coach-review instructional approach on caregiver use of language support strategies and children’s expressive language skills. *Journal of Speech, Language, and Hearing Research*, *57*(5), 1851–1869.

Romski, M., Adamson, L., Cheslock, M., & Sevcik, R. (2000). Parent Perception of Language Development (PPOLD). *Unpublished Manuscript, Georgia State University, Atlanta*.

Sandwell Primary Care Trust. (2012). *Wellcomm: A speech and language toolkit for the early years*. Granada/NFER Nelson UK.

Sawyer, B., Hindman, A., Smith, J., Hammer, C. S., & Santoro, J. (2025). Parents Plus: A Parent-Implemented Intervention for Preschool Children With Developmental Language Disorders. *Language, Speech, and Hearing Services in Schools*, *56*(1), 177–193. https://doi.org/10.1044/2024_LSHSS-24-00042

Semel, E., Wiig, E., & Secord, W. (2006). *Child evaluation of language fundamentals-preschool UK*. Oxford: Pearson Assessment.

Senent-Capuz, N., Baixauli-Fortea, I., & Moret-Tatay, C. (2021). Parent-Implemented Hanen Program It Takes Two to Talk(R): An Exploratory Study in Spain. *International Journal of Environmental Research and Public Health*, *18*(15). https://doi.org/10.3390/ijerph18158214

Snowling, M. J., Stothard, S. E., Clarke, P., Bowyer-Crane, C., Harrington, A., Truelove, E., Nation, K., & Hulme, C. (2009). *YARC York Assessment of Reading for Comprehension Passage Reading.* GL Publishers.

Snowling, M. J., Stothard, S. E., & Clarke, P. J. (2012). *York assessment of reading for comprehension: Complete set*. GL assessment.

Subba Rao, T. (1992). Manual on developing communication skills in mentally retarded persons. *NIMH, Secunderabad*.

Suttora, C., Zuccarini, M., Aceti, A., Corvaglia, L., Guarini, A., & Sansavini, A. (2021). The Effects of a Parent-Implemented Language Intervention on Late-Talkers’ Expressive Skills: The Mediational Role of Parental Speech Contingency and Dialogic Reading Abilities. *Frontiers in Psychology*, *12*, 723366. https://doi.org/10.3389/fpsyg.2021.723366

Tang, L., Zhao, J. Z., He, T. Y., Xu, L., He, X. J., Huang, S., & Hao, Y. (2024). Effect of online parent training in promoting language development of children with language delay in Hubei province, China. *International Journal of Language & Communication Disorders*. https://doi.org/10.1111/1460-6984.12945

Thomas-Stonell, N., Robertson, B., Walker, J., Oddson, B., Washington, K., & Rosenbaum, P. (2012). FOCUS\copyright: Focus on the outcomes of communication under six. *Holland Bloorview Kids Rehabilitation Hospital*.

Vally, Z., Murray, L., Tomlinson, M., & Cooper, P. J. (2015). The impact of dialogic book‐sharing training on infant language and attention: A randomized controlled trial in a deprived South African community. *Journal of Child Psychology and Psychiatry*, *56*(8), 865–873. https://doi.org/10.1111/jcpp.12352

Vrinda, R., & Kunnath, S. K. (2023). A Pilot Study to Evaluate the Effectiveness of Parent mediated Group Intervention Program for Developmental Language Disorders. *Clinical Archives of Communication Disorders*, *8*(2), 47–56.

Wake, M., Levickis, P., Tobin, S., Gold, L., Ukoumunne, O. C., Goldfeld, S., Zens, N., Le, H. N. D., Law, J., & Reilly, S. (2015). Two-Year Outcomes of a Population-Based Intervention for Preschool Language Delay: An RCT. *Pediatrics*, *136*(4), e838–e847. https://doi.org/10.1542/peds.2015-1337

Zhang, X., & Li, J. (1994). The revise of Gesell Developmental Scale on 31/2–6 years of age in Beijing. *Zhongguo Dang Dai Er Ke Za Zhi*, *2*, 148–150.

Zimmerman, I., Steiner, V., & Pond, R. (2002). *edition 4. Preschool language scale*. San Antonio, TX. Harcourt Assessment.

Zuccarini, M., Suttora, C., Bello, A., Aceti, A., Corvaglia, L., Caselli, M. C., Guarini, A., & Sansavini, A. (2020). A Parent-Implemented Language Intervention for Late Talkers: An Exploratory Study on Low-Risk Preterm and Full-Term Children. *International Journal of Environmental Research and Public Health*, *17*(23). https://doi.org/10.3390/ijerph17239123
